# Progranulin deficiency associates with postmenopausal osteoporosis via increasing ubiquitination of estrogen receptor α

**DOI:** 10.1016/j.gendis.2024.101221

**Published:** 2024-01-28

**Authors:** Guangfei Li, Aifei Wang, Wei Tang, Wenyu Fu, Qingyun Tian, Jinlong Jian, Michal Lata, Aubryanna Hettinghouse, Yuanjing Ding, Jianlu Wei, Xiangli Zhao, Mingyong Wang, Qirong Dong, Chuanju Liu, Youjia Xu

**Affiliations:** a Department of Orthopedics, Second Affiliated Hospital of Soochow University, Suzhou, Jiangsu 215004, China; b Department of Orthopaedic Surgery, New York University Grossman School of Medicine, New York, NY 10003, USA; c Osteoporosis Institute of Soochow University, Suzhou, Jiangsu 215004, China; d Department of Pathogenic Biology, School of Basic Medical Sciences, Shandong University, Jinan, Shandong 250012, China; e Department of Orthopaedics and Rehabilitation, Yale University School of Medicine, New Haven, CT 06510, USA; f Department of Orthopaedic Surgery, Jinan Central Hospital Affiliated to Shandong First Medical University, Jinan, Shandong 250013, China; g Department of Orthopaedic Surgery, Qilu Hospital of Shandong University, Jinan, Shandong 250012, China; h Murui Biological Technology Co., Ltd., Suzhou Industrial Park, Suzhou, Jiangsu 215123, China; i Department of Cell Biology, New York University Grossman School of Medicine, New York, NY 10016, USA

**Keywords:** Estrogen receptor α, Osteoclastogenesis, Postmenopausal osteoporosis, Progranulin, Ubiquitination

## Abstract

Estrogen deficiency is considered the most important cause of postmenopausal osteoporosis. However, the underlying mechanism is still not completely understood. In this study, progranulin (PGRN) was isolated as a key regulator of bone mineral density in postmenopausal women through high throughput proteomics screening. In addition, PGRN-deficient mice exhibited significantly lower bone mass than their littermates in an ovariectomy-induced osteoporosis model. Furthermore, estrogen-mediated inhibition of osteoclastogenesis and bone resorption as well as its protection against ovariectomy-induced bone loss largely depended on PGRN. Mechanistic studies revealed the existence of a positive feedback regulatory loop between PGRN and estrogen signaling. In addition, loss of PGRN led to the reduction of estrogen receptor α, the important estrogen receptor involved in estrogen regulation of osteoporosis, through enhancing its degradation via K48-linked ubiquitination. These findings not only provide a previously unrecognized interplay between PGRN and estrogen signaling in regulating osteoclastogenesis and osteoporosis but may also present a new therapeutic approach for the prevention and treatment of postmenopausal osteoporosis by targeting PGRN/estrogen receptor α.

## Introduction

Osteoporosis is considered one of the most serious chronic diseases of the present century.^1^ Postmenopausal osteoporosis is the most prevalent type of osteoporosis. Approximately one in three women may suffer from osteoporotic fractures during their lifetime.^2^ Patients with postmenopausal osteoporosis-related fractures are also at a higher risk of subsequent fractures, along with increased morbidity and premature mortality,^3^ leading to a heavy economic burden for both patients and society. Increased bone resorption in postmenopausal women is caused by a lack of estrogen and can be prevented by estrogen replacement therapy.^4^ However, the underlying mechanisms are not completely understood. In contrast, high doses of estrogen have been shown to exert anabolic skeletal effects in rodents and postmenopausal women.^5^^,^^6^

Progranulin (PGRN) is a secretory 593-amino acid growth factor-like molecule that is widely expressed in different cells, including osteoclasts, osteoblasts, and chondrocytes.^7^, ^8^, ^9^, ^10^ PGRN has been reported to inhibit inflammatory pathways that suppress osteoclastogenesis and prevent bone loss.^11^, ^12^, ^13^ Additionally, recombinant PGRN has been shown to enhance bone regeneration under both physiological and diabetic conditions.^14^, ^15^, ^16^ Furthermore, a recent study demonstrated that risedronate, a bisphosphonate drug that has been widely used in the treatment of osteoporosis, induces the expression and secretion of PGRN in osteoblastic cells, leading to the proliferation and survival of these cells.^17^ A recent study reported a significant positive correlation between serum PGRN levels and hip bone mineral density (BMD) in obese individuals.^18^ Nevertheless, the association between PGRN and BMD in postmenopausal women has yet to be determined. This study aimed to determine the association between bone PGRN expression and BMD in postmenopausal women as well as the underlying mechanisms.

## Materials and methods

### Study population

This study complies with the ethical regulations for work with human bone tissue samples and was approved under JD-LK-2020-027-01 by the Second Affiliated Hospital of Soochow University. All patients who were diagnosed with one-sided femoral neck fracture and needed to be treated with hip replacement surgery were recruited from the Department of Orthopaedics, Second Affiliated Hospital of Soochow University, China, between 2012 and 2018. The inclusion criterion was postmenopausal women with one-sided femoral neck fracture occurring for the first time due to low-energy injury that required artificial joint replacement. The patients with chronic liver and kidney diseases, metabolic disease, tumor, and hematological disease; long-term treatment with glucocorticoids, estrogen, calcitonin, and bisphosphonates; bilateral hip fractures; and hip pathological fractures were excluded. Each participant was completely informed of the study protocol and signed an informed consent form before enrollment.

### Anthropometric measurement

Weights and heights were measured and recorded with participants wearing light clothes without shoes. Body mass index was calculated using the “weight (kg)/height^2^ (m^2^)" equation.

### Laboratory measurements

All blood samples were collected between 8:00 a.m. and 10:00 a.m. from patients with an 8–12 h fast. To collect serum, blood samples were centrifuged at 3000 rpm for 10 min. Serum parathyroid hormone levels were measured using an ELISA kit (Beckman Coulter Life Sciences, Inc.). Serum 25(OH) vitamin D, type I procollagen amino-terminal peptide, and β-type I collagen carboxy-terminal peptide were measured using diagnostic ELISA kits (Roche, Inc.). Hemoglobin was measured using a hematology automated analyzer (Sysmex, Inc.). Serum levels of calcium, phosphorus, uric acid, albumin, alkaline phosphatase (ALP), and C-reactive protein were measured using the Cobas 8000 modular analyzer (Roche, Inc.).

### BMD measurement

Dual-energy X-ray absorptiometry was used to measure BMD at the lumbar spine and the hip of the non-injured side three days after replacement surgery using GE Lunar DPX. We used the hip BMD of the non-injured side to represent the hip BMD of the injured side, where the hip bone was collected during the surgery. Based on the standards of the World Health Organization, normal bone mass was defined as BMD ≥ −1 standard deviation (SD), osteopenia as BMD between −1 and −2.5 standard deviation, and osteoporosis as BMD ≤ −2.5 standard deviation.

### Acquisition of the femoral head specimens and mass spectrometry

**Figure 1:**
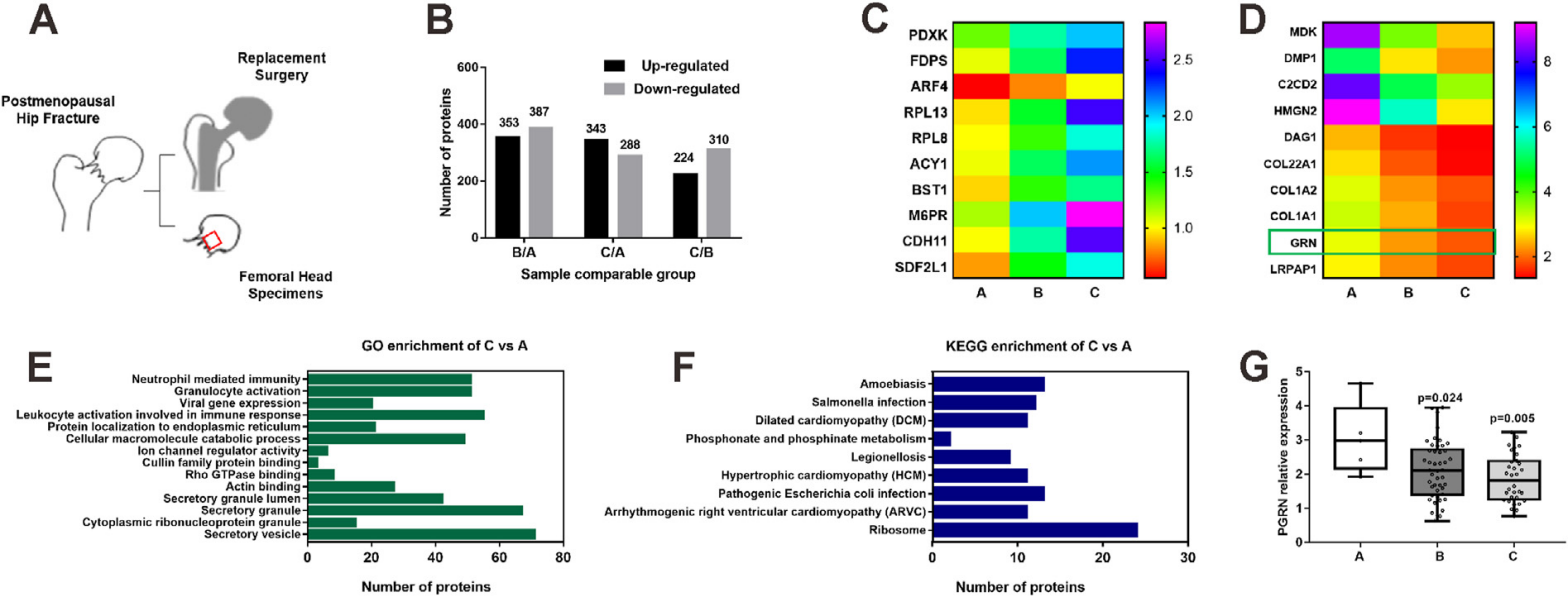
Hip bone PGRN expression positively correlates with hip BMD. **(A)** The schematic diagram illustrating the acquisition of femoral head specimens. The red area indicated where mass spectrometry was performed. **(B)** Number of up-regulated and down-regulated proteins between every two groups. **(C, D)** Top 10 consecutive up-regulated (C) and down-regulated (D) proteins from group A to group C. **(E)** GO enrichment analysis of group C versus group A. **(F)** KEEG enrichment analysis of group C versus group A. **(G)** Relative PGRN expression in the hip bone tissue in each group. Data are presented as box and whisker plots showing the median, minimum to maximum. Osteopenia (B) and Osteoporosis (C) groups were compared with the Normal (A) group using a Dunnett's test, with a significance level set to 0.05 (*P* < 0.05). PGRN, progranulin; BMD, bone mineral density.

Due to hip fragility fractures, all patients underwent hip replacement surgery, such as hemiarthroplasty or total arthroplasty. With the patients' consent and as per the study protocol, the femoral head of the injured side was removed during the surgery (Fig. 1A). The red area indicated the area from where the bone samples were sent for mass spectrometry. The bone samples were decalcified in 1.2 M HCl at 4 °C overnight, and the resultant supernatant collected by centrifugation was marked as extract A. The remaining bone tissues were rinsed with deionized water at 4 °C, and immersed in a solution containing 100 mM Tris, 6M guanidine-HCl (pH 7.4), and protease inhibitor for 72 h. The supernatant was collected by centrifugation and used as extract B. The remaining bone tissues were immersed for 72 h in 100 mM Tris, 6M guanidine-HCl, and 0.5 M tetrasodium ethylenediaminetetraacetic acid solution at 4 °C, and the supernatant collected was used as extract C. Finally, the remaining bone tissues were further immersed in 6M HCl solution at 4 °C overnight, the supernatant was collected and marked as extract D, and the extracts (A, B, C, and D) were combined and precipitated with acetone at −20 °C. After washing with acetone three times, the precipitate was re-dissolved with 8 M urea. Following centrifugation for 10 min, the supernatant was considered as a protein solution and subjected to mass spectrometry analysis. After trypsin digestion, TMT labeling, and high-performance liquid chromatography fractionation, mass spectrometry was performed by PTM Biolabs (contract number PTM20170929C02; Hangzhou, China). All MS/MS spectra were collected with the following settings: resolution, 30,000; AGC target, 5 × 10^4^; maximum time, 4000 ions/s; fixed first mass, 100 *m*/*z*; and NCE, 28. The resulting MS/MS data were processed using the MaxQuant search engine (v.1.5.2.8). Tandem mass spectra were searched against the SwissProt human database concatenated with a reverse decoy database. False discovery rate thresholds used to filter out false positive protein, peptide, and modification sites were determined as 1 %.

### Gene ontology (GO) annotation (GOA) and enrichment analysis

The GO annotation proteome provided by the UniProt-GOA database (https://www.ebi.ac.uk/GOA/) was used to annotate the protein GO function. If some identified proteins were not annotated by the UniProt-GOA database, the InterProScan that allowed the proteins to be searched and annotated based on protein sequence was used instead. Further, proteins were classified by GOA according to three independent categories including biological process, cellular component, and molecular function. The two-tailed Fisher's exact test was used to determine the enrichment of differentially expressed proteins against all identified proteins for each category. An adjusted *P*-value less than 0.05 was considered to be statistically significant.

### KEGG pathway annotation and enrichment analysis

Kyoto Encyclopedia of Genes and Genomes (KEGG) pathway enrichment analysis was subsequently conducted to annotate signal pathways. Following the functional annotation of protein using the KEGG automatic annotation server, the results were then mapped to the KEGG pathway database with the KEGG mapper. The KEGG database was used to identify enriched pathways representing the enrichment of the differentially expressed proteins against all identified proteins by a two-tailed Fisher's exact test. The pathway with a corrected *P*-value less than 0.05 was considered statistically significant. These pathways were classified into hierarchical categories according to the KEGG database.

### Mice

Wild-type (WT) C57BL/6 mice were obtained from the Jackson Laboratory. PGRN-knockout (PGRN-KO) mice on a C57BL/6 genetic background were established and maintained at the laboratory.^11^ All mice used in the current study were age- and sex-matched. Twelve-week-old female mice were used for the ovariectomy (OVX)-induced osteoporosis model and 40-week-old female mice were used for the aged osteoporosis model. During OVX, the bilateral ovaries were removed from the dorsal approach. Sham operations were also performed to confirm the establishment of the OVX model. The WT OVX and PGRN-KO OVX mice were treated with 17β-estradiol pellets (customized from the Innovative Research of America, FL, US) at a dosage of 0.005 mg and release time of 21 days or placebo pellets (Innovative Research of America). Immediately after OVX surgery, 17β-estradiol pellets and placebo pellets were implanted into the lateral side of the neck under the skin of mice in the treatment and control groups, respectively; one pellet per animal. Three weeks after OVX and pellet implantation surgery, the mice were euthanized, the femurs were subjected to micro-CT scanning, and the tibias were subjected to histological analyses. All animal experiments were conducted in accordance with the institutional guidelines and approved by the IACUC of the New York University School of Medicine.

### Micro-computed tomography (CT) analysis

Micro-CT analysis was performed at New York University, as previously described.^15^ Briefly, after being fixed in 4% paraformaldehyde at room temperature for 24 h and washed three times with phosphate buffer saline solution (PBS), the femurs were stored in 70% alcohol before micro-CT processing. A Skyscan 1172 cone-beam scanner (Skyscan 1172) was used for tissue scanning with X-ray intensity, X-ray tube potential, and voxel size set at 145 mA, 55 kVp, and 10.5 μm^3^, respectively. The femoral long bone was scanned completely and used for analysis of the morphometric parameters of the bone quality on reconstructed transaxial datasets using Bruker-micro-CT CT-Analyzer (v. 1.13) software for various parameters, including total BMD, total volume (TV), bone volume (BV), BV/TV, trabecular bone thickness, trabecular bone number, trabecular bone space, cortical BMD, cortical thickness, and cortical area. The region of interest for trabecular bone within the distal femur was chosen between 0.10 mm and 1.35 mm proximal to the growth plate, excluding the cortical bone. The region of interest for cortical bone within the femur was defined to commence about 2.15 mm from the growth plate in the direction of the metaphysis. Segmentation was performed on 60 slices for each sample.

### Soft X-ray photography

Radiographs of aged mice's spines were recorded with a Faxitron X-ray machine (Wheeling, IL) at 5.0-kV over 6.0 s.

### Biomechanical testing

The femurs dissected free of remaining soft tissue were subjected to three-point bending tests using the MTS 858 Bionix Testing Machine (MTS 858 Mini Bionix, MTS Systems, Corp, Eden Prairie, Minnesota) at 2 mm/min until failure. The femur samples were immersed in normal saline at room temperature. The maximum load (N), maximum displacement (mm), and post-yield displacement (mm) data were collected from each test. The toughness (N/mm^2^) and stiffness (N/mm) were calculated by Dr. Oran D. Kennedy's laboratory at New York University.

### Histology

The mouse tibia bone tissues were fixed in 4% phosphate-buffered paraformaldehyde, decalcified with 10% ethylenediaminetetraacetic acid for two weeks, and embedded in paraffin. The sectioned tibia tissue was processed for TRAP staining^13^ and imaged using a Zeiss microscope. Hematoxylin-eosin staining was performed as reported previously.^19^^,^^20^ Osteoclast surface to bone surface (Oc.S/BS), osteoclast number per bone surface (Oc.N/BS), osteoblast surface to bone surface (Ob.S/BS), osteoblast number per bone surface (Ob.N/BS), osteoid volume/bone volume (OV/BV), osteoid surface/bone surface (OS/BS), osteoid thickness, and eroded surface/bone surface (ES/BS) were quantified using BIOQUANT OSTEO 2017 v17.2.60 software.

### Immunohistochemistry staining

Sections were prepared as described previously. After being deparaffinized and rehydrated and subsequently digested with 0.1% trypsin for 30 min and 0.25 U/mL chondroitinase ABC (Sigma–Aldrich) and 1 U/mL hyaluronidase (Sigma–Aldrich) for 60 min, respectively, at 37 °C, the tibia sections were then incubated with anti-ERα primary antibody (1:100, sc-514857) and anti-PGRN primary antibody (1:100, catalog sc-28928) at 4 °C overnight. Sections were stained with 0.5 mg/mL 3,3-diaminobenzidine in 50 mM Tris-Cl substrate (Sigma–Aldrich) to observe the chromogen and 1% methyl green or hematoxylin for counterstaining. Images were obtained using a Zeiss microscope.^13^

### Isolation and differentiation of bone marrow-derived macrophages (BMMs)

The bone marrow cells from the femurs and tibias were collected as described previously^16^ and cultured in α-MEM containing 10% fetal bovine serum and 10 ng/mL M-CSF (Biolegend) for macrophage differentiation for three days.

### Culture of primary mouse bone marrow-derived mesenchymal stromal/stem cells (BMSCs)

The bone marrow cells harvested from the long bones were grown in culture dishes for 3 h to allow attachment of adherent cells, and the dishes were then rinsed twice with PBS to remove the non-adherent cells. The medium was replaced every three days. After 12–15 days of culture, BMSCs were formed as described previously.^21^

### Estrogen treatment of cells

The Raw264.7 cells were obtained from the American Type Culture Collection. The BMMs and Raw264.7 cells were treated with 10 nM 17β-estradiol (Sigma, E-2758, Lot 122K1535) as per a previous study.^22^ The BMSCs were treated with 100 nM 17β-estradiol as per a previous study.^23^ The control group of the cell was treated with PBS. Fulvestrant (SelleckChem, ICI-182780, Catalog No. S1191) was used as an antagonist of the estrogen receptor (ER).

### Osteoclast differentiation and TRAP staining

To differentiate into osteoclasts, the Raw264.7 cells were cultured in α-MEM supplemented with 10% fetal bovine serum and 50 ng/mL RANKL (R&D Systems) for four days. The BMMs were obtained and cultured in α-MEM containing 10% fetal bovine serum, 10 ng/mL M-CSF, and 50 ng/mL RANKL with replenishment every two days for a total of four days. The differentiated cells were processed for TRAP staining and the number of TRAP-positive multinucleated cells with more than three nuclei were counted under microscopy, as previously described.^11^

### Resorption pit assay

Primary BMMs were re-seeded in Osteo Assay Surface 24-well plates (Corning, Corning, NY, USA) at a density of 2.5 × 10^4^ cells/well in α-MEM containing 10% fetal bovine serum, 10 ng/mL M-CSF, and 50 ng/mL RANKL for seven days. The medium was replaced every two days. At the end of differentiation, the medium was removed from the wells, and 100 μL of 10% bleach solution was added to the cells at room temperature for 5 min. The wells were then washed twice with distilled water and allowed to dry at room temperature for 3–5 h before observing individual pits or multiple pit clusters under a microscope. The ratio of the resorbed area to the total area was calculated using ImageJ software.

### Quantitative reverse transcription polymerase chain reaction (RT-PCR)

Total RNA was isolated using an RNeasy Mini Kit (Qiagen) according to the manufacture's instruction and reverse-transcribed into cDNA using a High-Capacity cDNA Reverse Transcription Kit (Applied Biosystems). qRT-PCR was performed with SYBR green in triplicates. The primer sequences for the genes of interest in mice are listed in Table S1. The mRNA levels were normalized to levels of the housekeeping gene *Gapdh* to obtain relative mRNA fold change.

### Immunoblotting

Total protein (50 μg) from lysed cultured cells was subjected to SDS-PAGE and subsequently transferred to nitrocellulose membranes (Bio-Rad). Membranes were incubated with anti-PGRN (1:500, Santa Cruz Biotechnology, sc-28928) or anti-ERα (1:500, Santa Cruz Biotechnology, sc-514857). Following incubation with specific secondary antibodies, the target protein was detected with an enhanced chemiluminescent substrate.

### ELISA assay

PGRN levels were measured in sera isolated from mice and cell culture supernatants, respectively, using an ELISA kit following the manufacturer's instructions (AdipoGen; catalog AG-45A-0019Y).

### Immunofluorescence staining

When treatment processing was complete, the cell culture medium was removed, and the cells were fixed in cold acetone/methanol (1:1). After being blocked with donkey serum for 30 min at room temperature, the samples were incubated with anti-PGRN primary antibody (Santa Cruz Biotechnology; catalog sc-28928, 1:50 dilution) at room temperature for 1 h. Then fluorescein isothiocyanate conjugated secondary donkey anti-rabbit antibody (Santa Cruz Biotechnology; catalog sc-2090, 1:100 dilution) was applied to the samples for 1 h. DAPI (4,6-diamidino-2-phenylindole) was used to stain the nuclei. Image software (Media Cybernetics, Rockville, MD, USA) was used to capture images under a Zeiss Axioscope A1 microscope.

### Determination of osteogenic differentiation

BMSCs were cultured in StemXvivo osteogenic medium (R&D Systems) for osteogenic differentiation. After two weeks of differentiation, both ALP activity and staining were performed as previously described.^24^ After three weeks of differentiation, cells were fixed with 3.7% formalin and stained with 2% Alizarin Red S (ARS, pH 4.2) for 10 min (Sigma). Bound ARS was dissolved in a 10% cetylpyridinium chloride monohydrate solution (pH 7.0). The absorbance was measured at 540 nm using a microplate reader.

### Co-immunoprecipitation

BMMs were lysed in radioimmunoprecipitation assay buffer containing protease inhibitors. The total protein (400 μg) was immunoprecipitated using an anti-ERα primary antibody (sc-514857), and the protein complexes were detected using anti-ubiquitin antibodies. Antibodies against ubiquitin (catalog 3936S), K48-linkage-specific polyubiquitin (catalog 8081S), and K63-linkage-specific polyubiquitin (catalog 5621S) were purchased from Cell Signaling Technology.

### Statistical analysis

Comparisons between two groups were performed by two-tailed unpaired Student's *t*-tests, and comparison between multiple groups was analyzed by one-way ANOVA with Bonferroni's and Dunnett's post hoc test. The level of statistical significance was set at *P* < 0.05.

## Results

### Hip bone PGRN expression positively correlates with hip BMD

To gain comprehensive quantitative maps of protein expression landscape in bones and their relation to postmenopausal osteoporosis, we performed high-resolution mass spectrometry analysis on bone samples collected from 83 postmenopausal women with one-sided hip fragility fractures. The brief baseline characteristics of patients were summarized in Table 1 and details were provided in Supplementary File 1. We distributed the patients into three groups according to their femoral neck T-scores: the normal (A) group was assigned T ≥ −1.0, the osteopenia (B) group was assigned −2.5 < T < −1.0, and the osteoporosis (C) group was assigned T ≤ −2.5 (Table 1). Detailed information among the three groups including *F* value and *p* value about the baseline characteristics of patients was provided in Table S2. The osteopenia and osteoporosis groups had similar age, height, weight, and body mass index (*p* > 0.05) as the normal group. The greater trochanter BMD and total hip BMD were consistent with femoral neck BMD, while lumbar BMD was inconsistent with hip BMD. The osteopenia group had higher hemoglobin levels than those in the normal group (*P* < 0.05), while the osteoporosis group had similar hemoglobin levels as the normal group (*P* > 0.05). There were no significant differences in the serum levels of parathyroid hormone, vitamin D, calcium, phosphorus, uric acid, albumin, ALP, C-reactive protein, type I procollagen amino-terminal peptide, and β-type I collagen carboxy-terminal peptide in osteopenia and osteoporosis groups, when compared with the normal group (*P* > 0.05).

**Table 1:** Baseline characteristics of all the sample's corresponding clinical information by group.

Variables	Normal (A)	Osteopenia (B)	Osteoporosis (C)
Femoral neck T-score	*T* ≥ −1.0	−2.5 < T < −1.0	*T* ≤ −2.5
**Samples information**			
Total number of samples	5	46	32
Ordinal sample numbers	S1, S19, S46, S49, S60	S2, S5-8, S10–14, S16–17, S20–25, S27–29, S33, S37–38, S40–41, S43, S45, S50, S53, S55–56, S58, S62–63, S67, S69–70, S76–77, S80–81, S84, S92-94	S15, S18, S26, S30–31, S34, S36, S39, S42, S47, S51–52, S54, S57, S59, S64, S66, S71–75, S78, S82, S85–91, S95
**Anthropometry**			
Age (years)	79.20 ± 13.83	73.04 ± 7.97^#^	80.87 ± 6.81^#^
Height (cm)	156.00 ± 4.18	156.63 ± 4.97^#^	153.19 ± 4.30^#^
Weight (Kg)	49.00 ± 7.38	56.66 ± 8.16^#^	48.33 ± 7.49^#^
BMI (Kg/cm^2^)	20.21 ± 3.49	23.07 ± 3.01^#^	20.58 ± 3.24^#^
**Bone densitometry**			
Femoral neck T-score	0.46 ± 1.03	−1.81 ± 0.41∗∗∗	−3.23 ± 0.65∗∗∗
Femoral neck BMD (g/cm^2^)	0.98 ± 0.12	0.72 ± 0.55∗∗∗	0.55 ± 0.83∗∗∗
Lumbar T-score	−1.58 ± 0.92	−1.76 ± 1.10^#^	−2.68 ± 0.94∗
Lumbar BMD (g/cm^2^)	0.92 ± 0.11	0.90 ± 0.14^#^	0.79 ± 0.11∗
Greater trochanter BMD (g/cm^2^)	0.82 ± 0.37	0.61 ± 0.06∗∗∗	0.47 ± 0.08∗∗∗
Total hip BMD (g/cm^2^)	0.93 ± 0.09	0.76 ± 0.09∗∗∗	0.58 ± 0.09∗∗∗
**Biochemistry characteristics**			
PTH (pg/ml)	55.40 ± 20.51	72.69 ± 76.57^#^	66.90 ± 37.25^#^
Vitamin D (nmol/L)	32.04 ± 10.91	36.63 ± 10.84^#^	29.51 ± 7.10^#^
Hb (g/L)	104.40 ± 18.20	123.11 ± 14.49∗	115.44 ± 15.23^#^
Serum calcium (mmol/L)	2.18 ± 0.11	2.22 ± 0.13^#^	2.17 ± 0.12^#^
Serum phosphorus (mmol/L)	1.03 ± 0.21	1.09 ± 0.21^#^	1.08 ± 0.21^#^
Serum uric acid (μmol/L)	339.80 ± 55.76	272.63 ± 108.12^#^	292.22 ± 105.28^#^
ALB (g/L)	39.08 ± 5.82	40.23 ± 3.21^#^	37.43 ± 4.30^#^
ALP (U/L)	86.60 ± 22.51	80.67 ± 22.57^#^	82.25 ± 26.37^#^
CRP (mg/L)	25.78 ± 33.41	30.17 ± 30.42^#^	39.62 ± 29.94^#^
PINP (ng/mL)	64.46 ± 48.13	58.18 ± 44.37^#^	59.38 ± 29.54^#^
β-CTX (pg/ml)	536.66 ± 130.85	503.80 ± 357.18^#^	698.71 ± 378.13^#^

BMI, body mass index; BMD, bone mineral density; PTH, parathyroid hormone; Hb, hemoglobin; ALB, albumin; ALP, alkaline phosphatase; CRP, C-reactive protein; PINP, type I procollagen amino-terminal peptide; β-CTX, β-type I collagen carboxy-terminal peptide.

Osteopenia (B) and Osteoporosis (C) groups were compared with the Normal (A) group using a Dunnett's test, with significance noted below *P* < 0.05. ^#^*P* > 0.05 versus Normal (A); ∗*P* < 0.05 versus Normal (A); ∗∗*P* < 0.01 versus Normal (A); ∗∗∗*P* < 0.001 versus Normal (A).

During the replacement surgery, the femoral heads were collected (Fig. 1A). The red area in Figure 1A indicated the location on the bone where were analyzed by mass spectrometry. Given that the red area fit the femoral neck most, this accounted for the grouping by femoral neck T-score. Proteome analysis resulted in the identification of 3743 proteins and quantification of 3280 proteins (The data that support the findings of this study have been deposited to the ProteomeXchange Consortium via the iProX repository with the data set identifier PXD0004943000). Group B had 353 up-regulated and 387 down-regulated proteins than group A; group C had 343 up-regulated and 288 down-regulated proteins than group A; and group C had 224 up-regulated and 310 down-regulated proteins than group B (Fig. 1B). The top 10 up-regulated and down-regulated proteins in groups A–C are shown in Figure 1C and D. GO enrichment analysis of the group C versus group A revealed that secretory vesicles and secretory granules were among the most significantly enriched GO terms (Fig. 1E). KEGG enrichment analysis of the group C versus group A revealed that ribosome and pathogenic *Escherichia coli* infection were among the most significantly enriched pathways (Fig. 1F). The GO and KEGG enrichment analyses of the group C versus group B, and group B versus group A are shown in Figure S1.

Of note, PGRN was one of the top 10 down-regulated proteins in groups B and C with a decrease in the hip BMD in postmenopausal women as compared with group A with normal hip BMD (Fig. 1D), which is consistent with the observation that expression of PGRN associates with BMD in obese individuals.^18^ Furthermore, we observed that the hip bone PGRN expression was positively associated with hip BMD (*F* = 4.637; *P* = 0.012) (Fig. 1G). The ratio of PGRN relative expression in group B to group A was 0.694 (*P* = 0.024), and that in group C to group A was 0.617 (*P* = 0.005).

### PGRN deficiency exacerbates bone loss in OVX-induced and aged osteoporosis model

**Figure 2:**
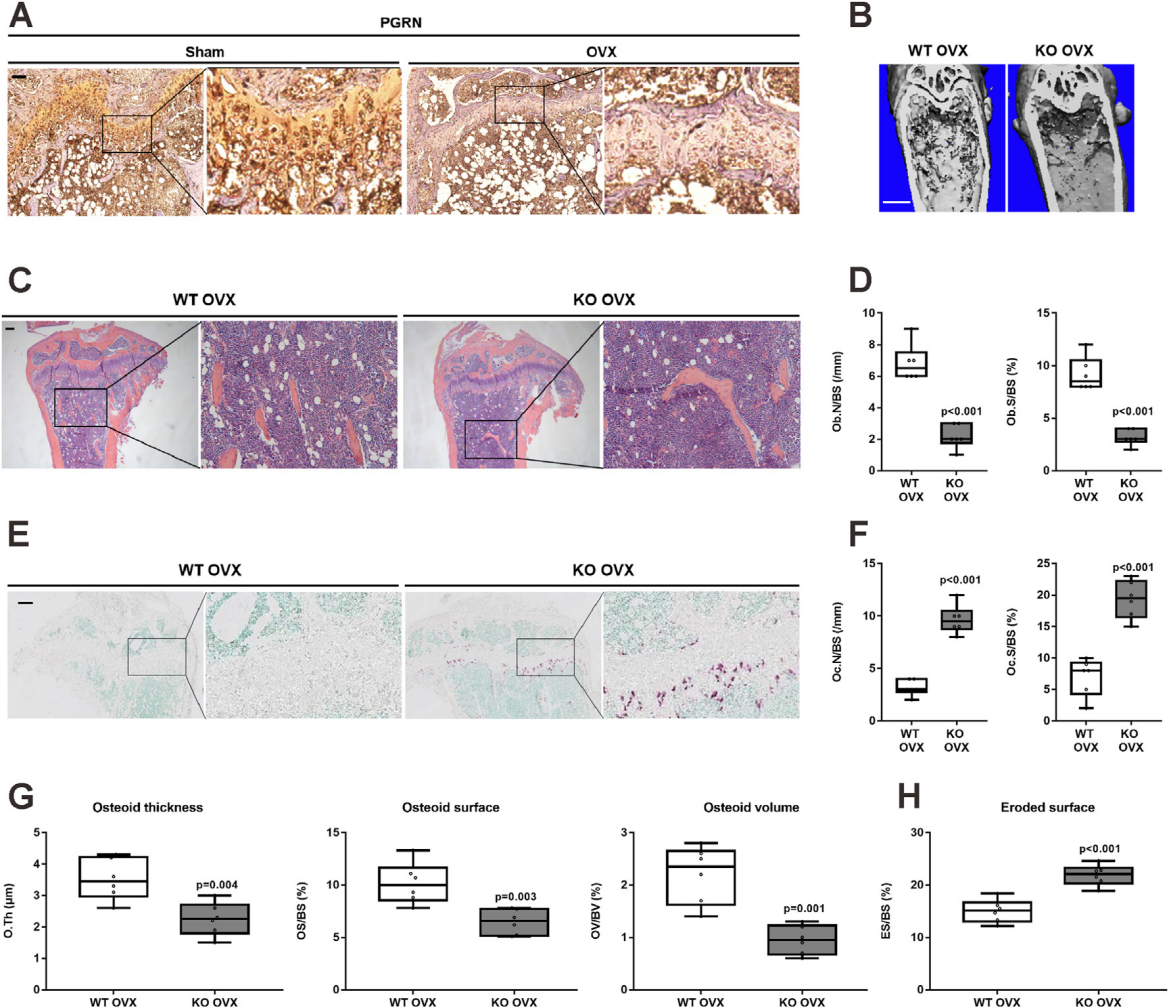
PGRN deficiency exacerbates bone loss in the OVX-induced osteoporosis model. **(A)** Immunohistochemistry staining of PGRN in tibia tissue sections of sham and OVX WT mice. Scale bar: 100 μm. **(B)** Coronal micro-CT images of the distal femur from 12-week-old OVX WT and PGRN-KO mice. Scale bar: 1 mm. **(C, D)** Hematoxylin-eosin staining (C) and quantification of Ob.N/BS and Ob.S/BS (D) in the proximal tibia of OVX 12-week-old WT and PGRN-KO mice. Scale bar: 200 μm. Two-tailed unpaired Student's *t*-tests. **(E, F)** TRAP staining (E) and quantification of Oc.N/BS and Oc.S/BS (F) in the proximal tibia of 12-week-old OVX WT and PGRN-KO mice. Scale bar: 200 μm. Two-tailed unpaired Student's *t*-tests. **(G)** Quantification of O.Th, OS/BS, and OV/BV in the proximal tibia sections of 12-week-old OVX WT and PGRN-KO mice. Two-tailed unpaired Student's *t*-tests. **(H)** Quantification of ES/BS in the proximal tibia sections of 12-week-old OVX WT and PGRN-KO mice. Two-tailed unpaired Student's *t*-tests. *n* = 6 mice per group. Data are presented as box and whisker plots showing the median, minimum to maximum. PGRN, progranulin; OVX, ovariectomy; Oc. N, osteoblast number; BS, bone surface; Oc.S, osteoclast surface; O.Th, osteoid thickness; OS, osteoid surface; OV, osteoid volume; BV, bone volume; ES, eroded surface.

The finding that PGRN was decreased in the bone of patients with osteopenia and osteoporosis led us to examine the levels of bone PGRN in OVX mice and found that PGRN was also decreased compared with that in sham-operated mice (Fig. 2A). As the bone PGRN expression was positively associated with BMD in postmenopausal women, we next investigated whether PGRN-deficient mice exhibited lower bone mass. First, no significant differences were observed in the bone mass between 12-week-old female WT and PGRN-KO mice (data not shown). However, PGRN deficiency enhanced OVX-induced bone loss in 12-week-old female mice (Fig. 2B). Additionally, hematoxylin-eosin staining revealed significantly lower trabecular bone number (Fig. 2C) and decreased Ob.N/BS and Ob.S/BS (Fig. 2D) in PGRN-deficient OVX mice than those in WT OVX littermates. TRAP staining revealed a significantly larger number of osteoclasts (Fig. 2E) and increased Oc.N/BS and Oc.S/BS (Fig. 2F) in the proximal tibia in PGRN-deficient OVX mice. Furthermore, PGRN-deficient OVX mice exhibited significantly decreased osteoid thickness, osteoid surface, osteoid volume, and increased eroded surface (Fig. 2G, H) than those in WT OVX littermates. In addition, at 40 weeks of age, PGRN-deficient female mice exhibited decreased bone mass in spines (Fig. S2A) and more severe bone loss, illustrated by lower trabecular bone mass relative to age-matched WT littermates (Fig. S2B). Consistently, TRAP staining revealed a significantly larger number of osteoclasts (Fig. S2C) and increased Oc.S/BS and Oc.N/BS (Fig. S2D) in the proximal tibia in PGRN-deficient aged mice than their WT littermates.

### Estradiol stimulates PGRN expression in both BMMs and Raw264.7 cells

**Figure 3:**
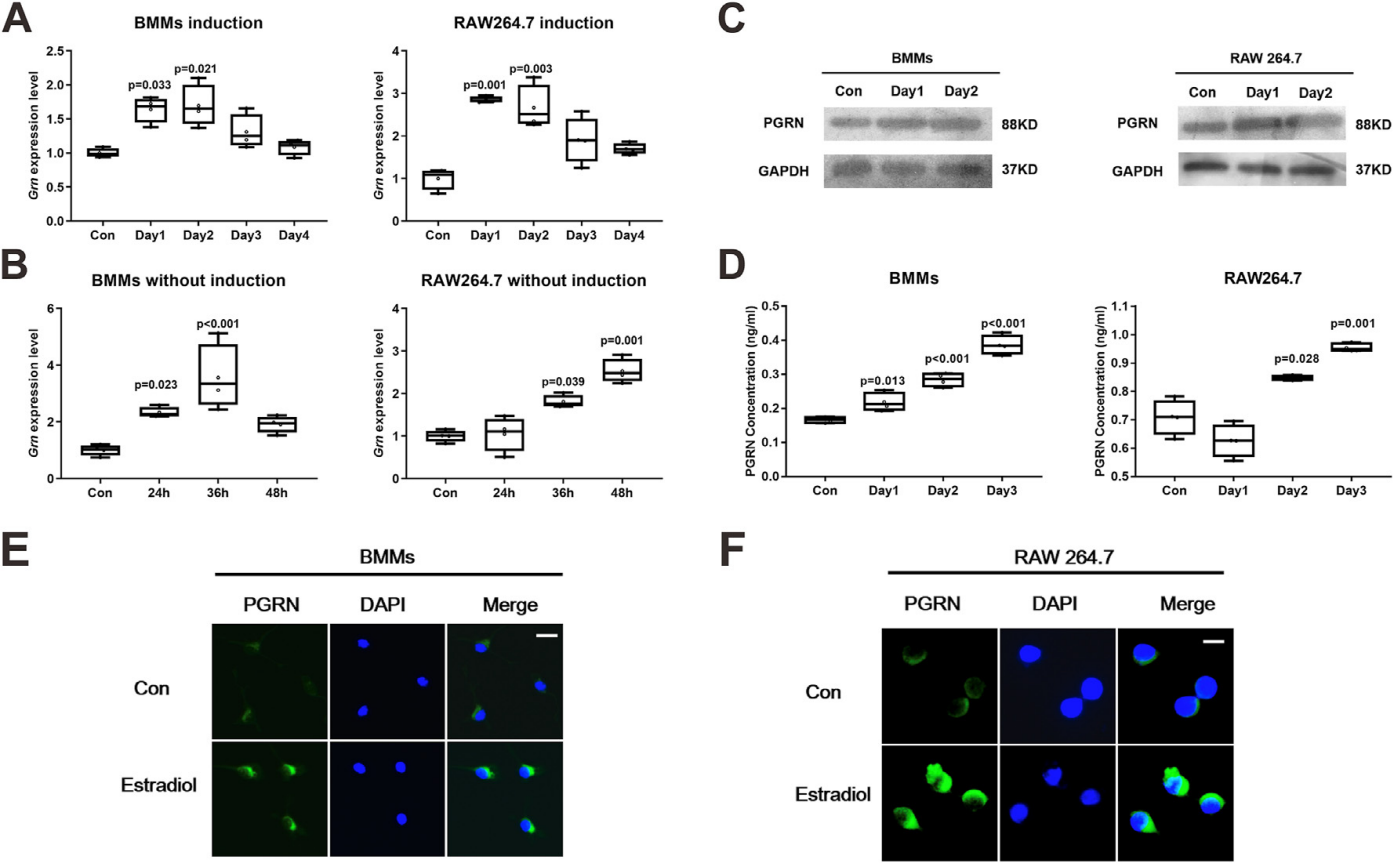
Estradiol stimulates PGRN expression in both BMMs and RAW 264.7 cells. **(A, B)** BMMs and RAW 264.7 cells were treated with estradiol (10 nM) for four days in osteoclastic differentiation medium (α-MEM with 10 ng/mL M-CSF and 50 ng/mL RANKL for BMMs and 50 ng/mL RANKL for RAW 264.7 cells) (A). BMMs and RAW 264.7 cells were treated with estradiol (10 nM) for two days in α-MEM (B). Cells were collected, and *Grn* mRNA levels were measured by real-time PCR. Estradiol-treated groups (*n* = 4 for each time point) were compared with the control group (*n* = 4) using a Dunnett's test, with a significance level set to 0.05 (*P* < 0.05). Data are presented as box and whisker plots showing the median, minimum to maximum. **(C)** BMMs and RAW 264.7 cells were treated with estradiol (10 nM) for two days, and PGRN protein levels were determined by immunoblotting. *n* = 3 biological replicates. **(D)** BMMs and RAW 264.7 cells were treated with estradiol (10 nM) for three days, and supernatant PGRN levels were determined by ELISA. Estradiol-treated groups (*n* = 4 for each time point) were compared with the control group (*n* = 4) using a Dunnett's test, with a significance level set to 0.05 (*P* < 0.05). Data are presented as box and whisker plots showing the median, minimum to maximum. **(E, F)** BMMs (E) and RAW 264.7 cells (F) were treated with or without estradiol (10 nM) for three days, and then cells were fixed and stained with PGRN primary antibody and donkey fluorescent secondary antibody. Scale bar: 25 μm. *n* = 3 biological replicates. “Con” indicates the control group treated with phosphate buffer saline solution. PGRN, progranulin; BMMs, marrow-derived macrophages.

In the absence of estrogen, PGRN-deficient mice exhibited lower bone mass than WT mice, suggesting that PGRN plays an important role in postmenopausal osteoporosis. Estrogen was reported to stimulate PGRN expression in human breast cancer cells.^25^ As estrogen is known to affect osteoclastogenesis and bone resorption,^26^ we then asked whether estrogen could stimulate PGRN expression in osteoclast precursors. In primary BMMs and Raw264.7 cells, estradiol increased the expression of *Grn,* the gene encoding PGRN, from day 1, in the presence of differentiation medium (*F* = 4.805 and *P* = 0.02 for BMMs with induction; *F* = 8.88 and *P* = 0.003 for Raw264.7 cells with induction) (Fig. 3A). Similar results were obtained in primary BMMs and Raw264.7 cells in the absence of differentiation medium (*F* = 6.188 and *P* = 0.018 for BMMs without induction; *F* = 14.754 and *P* = 0.001 for Raw264.7 cells without induction) (Fig. 3B). Both immunoblotting and immunofluorescence staining showed that estradiol increased PGRN expression in BMMs and Raw264.7 cells under osteoclastogenesis condition (Fig. 3C–E, F). Furthermore, as PGRN is a secreted protein^27^^,^^28^, its levels in the supernatant were measured by ELISA. The analysis suggested that treatment of osteoclast precursors with estradiol led to elevated PGRN levels in the cell culture supernatant (*F* = 77.432 and *P* < 0.001 for BMMs; *F* = 23.216 and *P* < 0.001 for Raw264.7 cells) (Fig. 3D). Taken together, the findings that estradiol induces PGRN expression in both BMMs and Raw264.7 cells indicate that PGRN may function as a downstream mediator of estrogen in osteoclastogenesis. Intriguingly, blockage of ERs with their antagonist fulvestrant largely abolished estradiol stimulated *Grn* expression in Raw264.7 cells with (*F* = 22.190; *P* = 0.002) (Fig. S3A) and without induction (*F* = 23.048; *P* = 0.002) (Fig. S3B).

### Loss of PGRN reduces estrogen-induced protection from bone loss after OVX in mice

**Figure 4:**
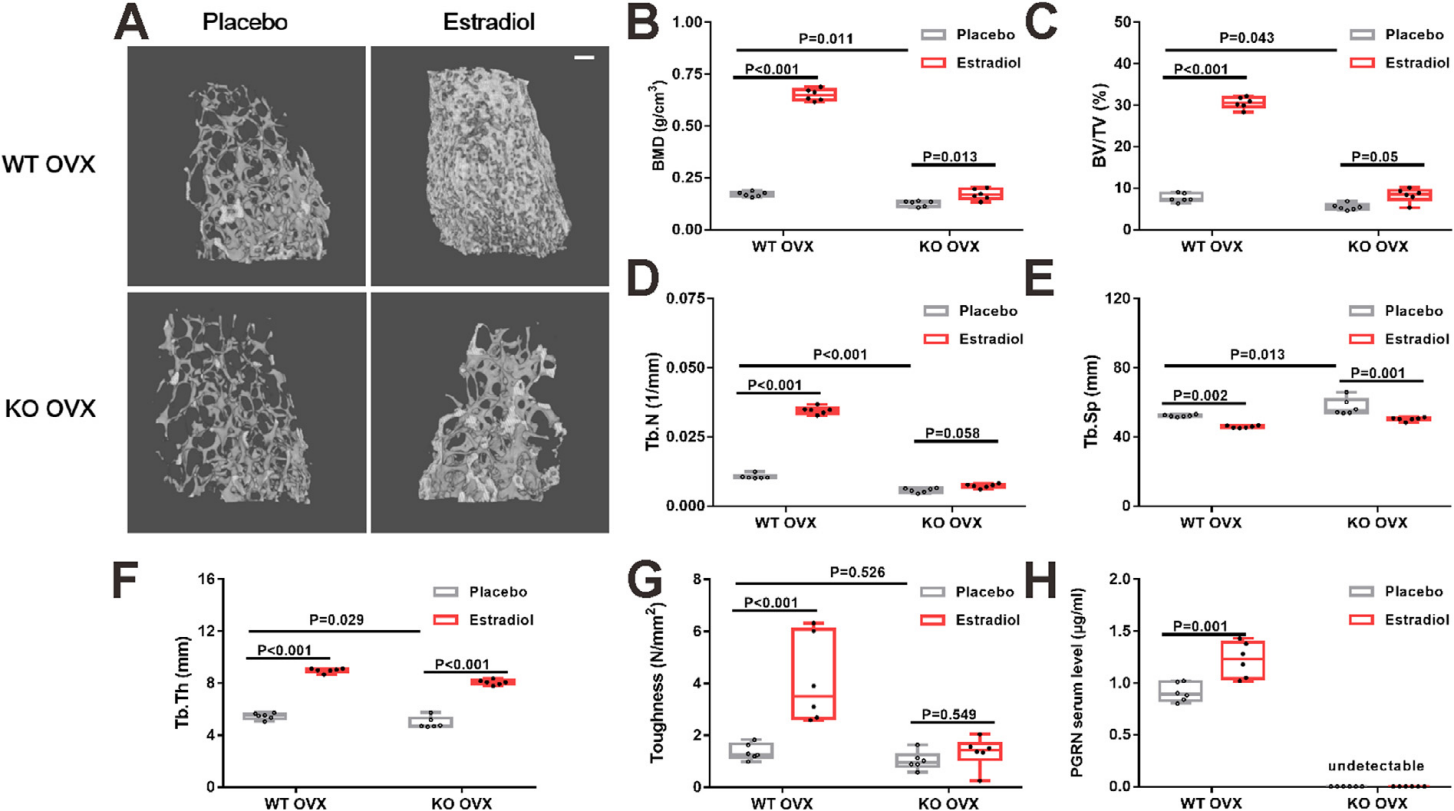
Estradiol's protection on cancellous bone loss after OVX is blunted in PGRN-KO mice. **(A)** Representative three-dimensional reconstruction of cancellous bone at distal femur of WT OVX mice and PGRN-KO OVX mice treated with estradiol or placebo. Scale bar: 250 μm. **(B–F)** Micro-CT assessment of cancellous BMD (B), BV/TV (C), Tb N (D), Tb Sp (E), and Tb Th (F) at distal femur of WT OVX mice and PGRN-KO OVX mice treated with estradiol or placebo. **(G)** Biomechanical testing of femur toughness of WT OVX mice and PGRN-KO OVX mice treated with estradiol or placebo. **(H)** Mouse serum PGRN concentration determined by ELISA. In A–H, *n* = 6 mice per group. In B–G, a significant difference was analyzed by one-way ANOVA with Bonferroni's post hoc test. In H, a significant difference was analyzed by a two-tailed unpaired Student's *t*-test, with a significance level set to 0.05 (*P* < 0.05). Data are presented as box and whisker plots showing the median, minimum to maximum. OVX, ovariectomy; PGRN, progranulin; BMD, bone mineral density; BV, bone volume; TV, total volume; Tb N, trabecular bone number; Tb Th, trabecular bone thickness; Tb Sp, trabecular bone space.

As estradiol stimulated PGRN expression in osteoclast precursor cells, we sought to determine whether the effect of estrogen on bone metabolism was mediated by PGRN. During OVX, the bilateral ovaries were excised from the dorsal approach, and sham operations were performed to confirm the successful establishment of the OVX model. Immediately after OVX, 17β-estradiol, and placebo pellets were implanted into the lateral side of the neck of the mice for steady and continuous release of the active ingredients. In line with Figure 2B, PGRN-KO OVX mice exhibited significantly lower trabecular bone mass than WT OVX mice (Fig. 4A). Furthermore, there was a significant decrease in trabecular BMD (*F* = 811.874; *P* < 0.001), BV/TV (*F* = 523.385; *P* < 0.001), trabecular bone number (*F* = 1159.838; *P* < 0.001), and trabecular bone thickness (*F* = 292.153; *P* < 0.001), and a significant increase in trabecular bone space (*F* = 21.196; *P* < 0.001) in the PGRN-KO OVX mice (Fig. 4B–F) than that in WT OVX mice. Treatment with 17β-estradiol significantly increased trabecular bone number, BMD, and BV/TV in the WT OVX mice, while these effects were largely lost in PGRN-KO mice (Fig. 4B–D). However, 17β-estradiol increased trabecular bone thickness and decreased trabecular bone space to the same extent in both WT OVX and PGRN-KO OVX mice (Fig. 4E, F). In contrast to its effects on trabecular bone in OVX mice, PGRN deficiency in OVX mice did not affect cortical bone as evidenced by comparable cortical bone mass, thickness, area, and BMD (Fig. S4). Treatment with 17β-estradiol significantly increased cortical thickness, area, and BMD in the WT OVX mice, while these effects were also blunted in PGRN-KO mice (Fig. S4). We also performed biomechanical testing and found that 17β-estradiol significantly increased the femur toughness (*F* = 14.283; *P* < 0.001), maximum load (*F* = 6.589; *P* = 0.003), maximum displacement (*F* = 13.243; *P* < 0.001), stiffness (*F* = 4.975; *P* = 0.01) and post-yield displacement (*F* = 15.223; *P* < 0.001) in WT OVX mice, while this effect was abolished in PGRN-KO OVX mice (Fig. 4G; Fig. S4). Additionally, the femur toughness tended to decrease in the PGRN-KO OVX mice than in WT OVX mice, although the difference did not reach statistical significance (Fig. 4G). Furthermore, we found that 17β-estradiol treatment significantly increased serum PGRN in WT OVX mice (Fig. 4H), which is in line with our *in vitro* results to confirm that 17β-estradiol stimulates PGRN expression in mice, which in turn increases bone mass.

### PGRN mediates estrogen-induced inhibition of osteoclast formation and bone resorption

**Figure 5:**
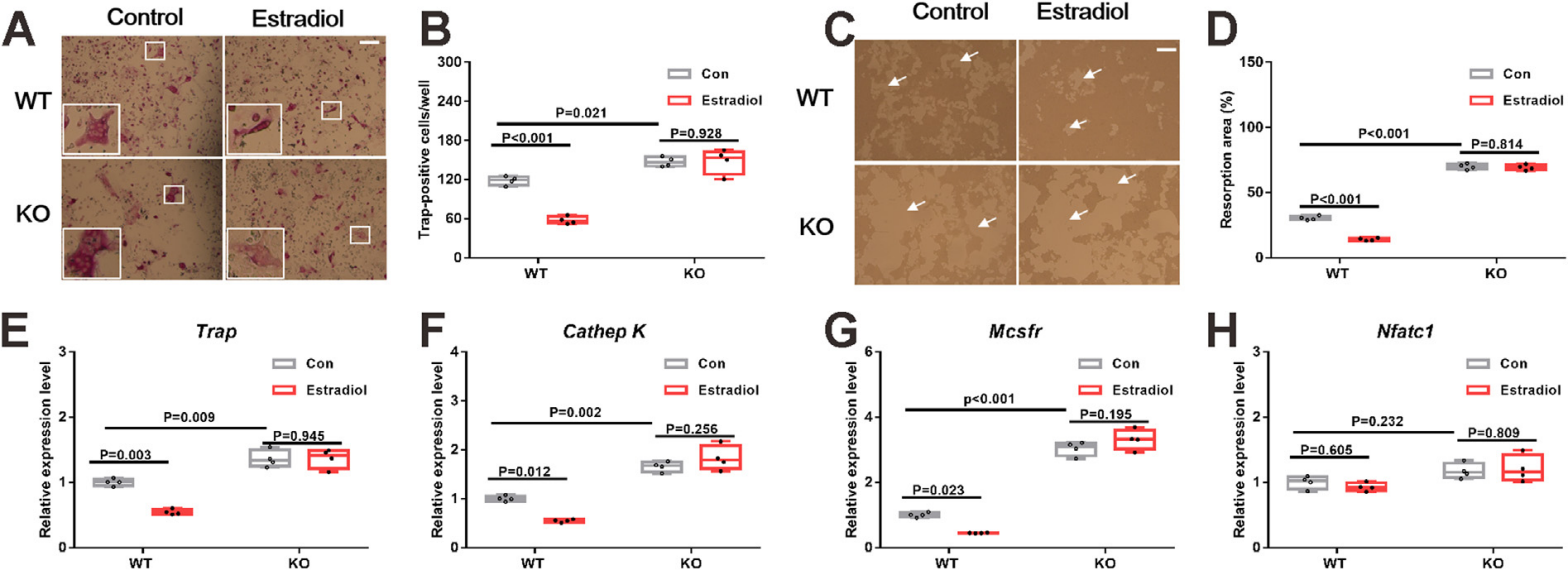
Estrogen's inhibition of osteoclast formation and bone resorption is abrogated in PGRN-KO BMMs. **(A, B)** Representative images (A) and quantified number (B) of TRAP-positive cells from both WT and PGRN-KO BMMs treated with or without estradiol (10 nM) under differentiation medium for four days. Insets shown are the enlarged area of white box regions in each image which highlight TRAP-positive osteoclasts. **(C, D)** Representative images of pits (C) and resorption area quantification (D) of both WT and PGRN-KO BMMs treated with or without estradiol (10 nM) under differentiation medium for seven days. White arrows indicate resorption areas. **(E**–**H)** mRNA expression of osteoclastic differentiation markers *Trap* (E), *Cathepsin K* (F), *Mcsfr* (G), and *Nfatc1* (H) of both WT and PGRN-KO BMMs treated with or without estradiol (10 nM) under differentiation medium for 48 h. In A and C, *n* = 4 biological replicates; scale bar: 100 μm. In B and D, a significant difference was analyzed by one-way ANOVA with Bonferroni's post hoc test. In E–H, *n* = 4 biological replicates; a significant difference was analyzed by one-way ANOVA with Bonferroni's post hoc test. “Con” indicates the control group treated with phosphate buffer saline solution. Data are presented as box and whisker plots showing the median, minimum to maximum. PGRN, progranulin; BMMs, marrow-derived macrophages.

The animal experiments suggested that PGRN was required, at least partially, for the effect of estrogen on bone metabolism; thus, we further examined whether it was due to the involvement of PGRN in osteoclastogenesis and/or osteoblastogenesis. First, the primary BMMs from PGRN-KO mice exhibited increased TRAP-positive cells (*F* = 55.585; *p* < 0.001) (Fig. 5A, B) and resorption areas (*F* = 927.945; *P* < 0.001) (Fig. 5C, D) during differentiation than BMMs from WT mice. The BMMs from PGRN-KO mice also displayed higher expression of osteoclastic gene markers including *Trap* (*F* = 26.955; *P* < 0.001) (Fig. 5E), *cathepsin K* (*F* = 35.561; *P* < 0.001) (Fig. 5F), and *Mcsfr* (*F* = 106.650; *P* < 0.001) (Fig. 5G) than BMMs from WT mice, except for *Nfatc1* (*F* = 2.017; *P* = 0.19) (Fig. 5H). Moreover, estradiol significantly inhibited osteoclastogenesis and bone resorption in the BMMs from WT mice, and this effect was largely blocked in PGRN KO BMMs (Fig. 5A–D). Accordingly, estradiol significantly inhibited the expression of *Trap*, *cathepsin K*, and *Mcsfr* in the BMMs from WT mice, but not in BMMs from PGRN-KO mice (Fig. 5E–G). Additionally, knockdown of *Grn* using siRNA in Raw264.7 cells recapitulated the effects of PGRN deficiency in the BMMs in terms of osteoclast differentiation and gene expression (*F* = 44.143 and *P* < 0.001 for Fig. S5C; *F* = 176.574 and *P* < 0.001 for Fig. S5D; *F* = 210.458 and *P* < 0.001 for Fig. S5E; *F* = 136.595 and *P* < 0.001 for Fig. S5F; *F* = 122.303 and *P* < 0.001 for Fig. S5G; *F* = 116.849 and *P* < 0.001 for Fig. S5H) (Fig. S5).

### Estradiol increases osteogenic differentiation of BMSCs from both WT and PGRN-KO mice

**Figure 6:**
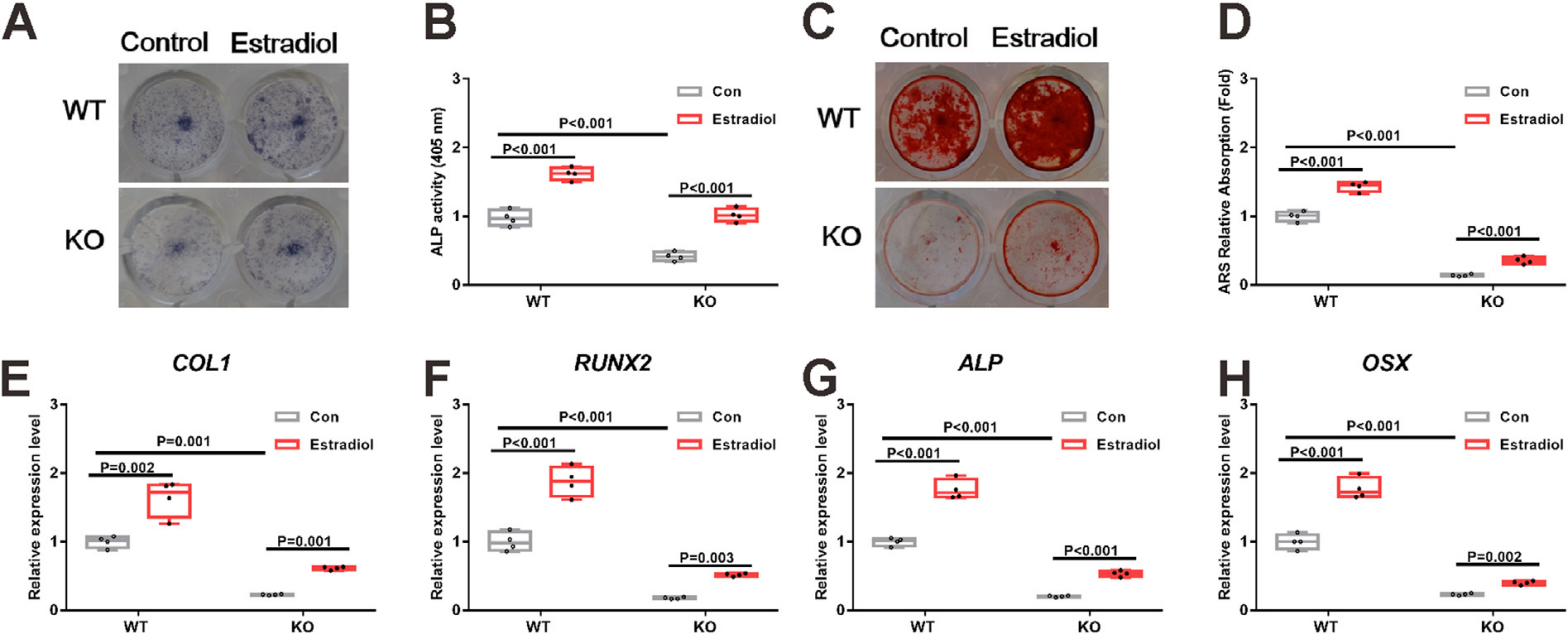
Estradiol increases osteogenic differentiation of BMSCs from both WT and PGRN-KO mice. **(A**–**D)** BMSCs from WT and PGRN-KO mice were treated with or without estradiol (100 nM) under osteogenic differentiation medium. ALP staining (A) and ALP activity (B) were performed at two weeks, ARS staining (C) was performed at three weeks, and the intensity (D) of ARS staining was quantified with 10 % CPC. In A and C, *n* = 4 biological replicates. In B and D, a significant difference was analyzed by one-way ANOVA with Bonferroni's post hoc test. **(E**–**H)** mRNA expression of osteogenic differentiation markers *Col 1* (E), *Runx 2* (F), *Alp* (G), and *Osx* (H) of WT and PGRN-KO BMSCs treated with or without estradiol (100 nM) under osteogenic differentiation medium for two weeks. In E–H, *n* = 4 biological replicates; a significant difference was analyzed by one-way ANOVA with Bonferroni's post hoc test. “Con” indicates the control group treated with phosphate buffer saline solution. Data are presented as box and whisker plots showing the median, minimum to maximum. PGRN, progranulin; BMSCs, bone marrow-derived mesenchymal stromal/stem cells.

Next, we asked whether PGRN mediated the effects of estrogen on osteogenic differentiation. We found that estradiol increased ALP staining (Fig. 6A), ALP activity (*F* = 106.319; *P* < 0.001) (Fig. 6B), and ARS staining (*F* = 428.119; *P* < 0.001) (Fig. 6C and D), along with mRNA expression of *Col 1* (*F* = 36.882; *P* < 0.001) (Fig. 6E), *Runx 2* (*F* = 128.071; *P* < 0.001) (Fig. 6F), *Alp* (*F* = 138.322; *P* < 0.001) (Fig. 6G), and *Osx* (*F* = 105.161; *P* < 0.001) (Fig. 6H) in BMSCs from both WT and PGRN-KO mice, indicating that the effect of estrogen on osteogenic differentiation may not be mediated by PGRN. Furthermore, we found that BMSCs from PGRN-KO mice exhibited decreased ALP staining (Fig. 6A), ALP activity (Fig. 6B), and ARS staining (Fig. 6C, D), along with decreased expression of osteogenic differentiation gene markers (Fig. 6E–H) than those from WT mice. We also examined the effects of PGRN knockout as well as recombinant PGRN on osteoblastogenesis and whether PGRN-mediated regulation of osteoblastogenesis depended on estrogen signaling, and found that blockage of estrogen signaling with ER inhibitor fulvestrant did not affect PGRN regulation of osteoblastogenesis (*F* = 32.383 and *P* < 0.001 for Fig. S6D) (Fig. S6).

### Loss of PGRN decreases ERα protein expression by increasing its ubiquitination

**Figure 7:**
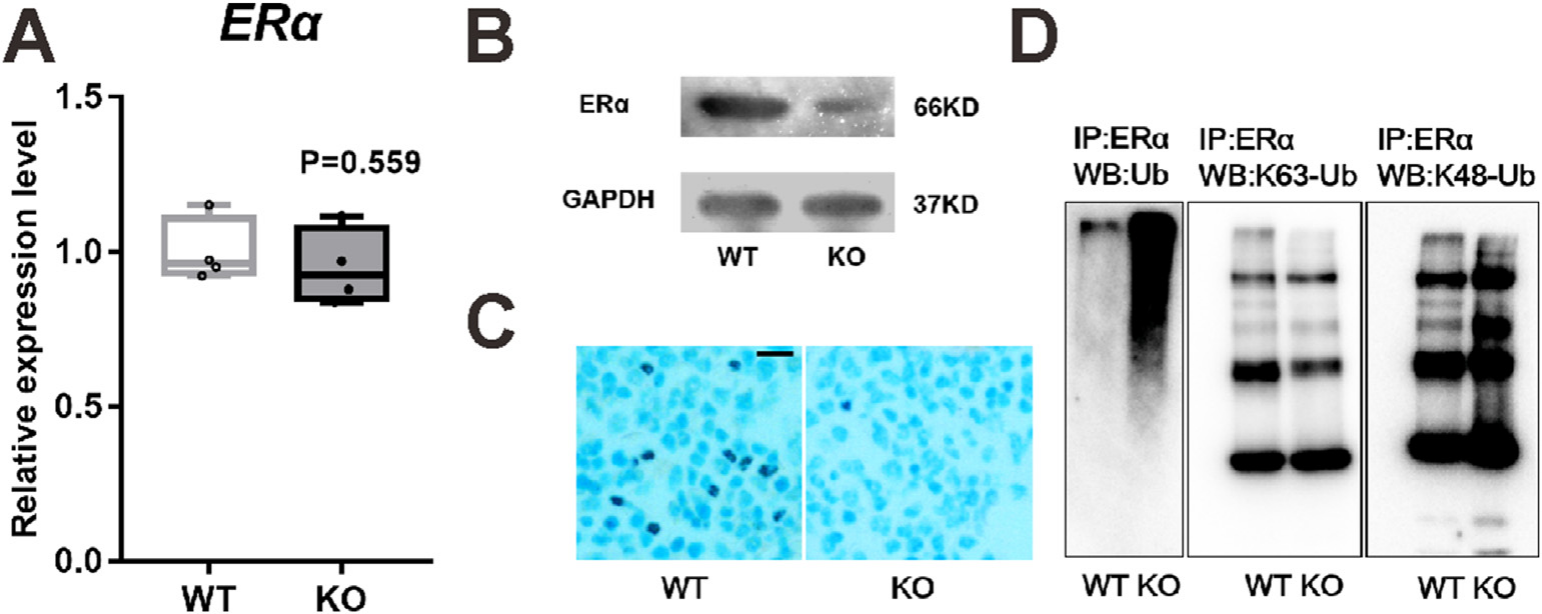
Loss of PGRN decreases ERα protein expression via increasing its ubiquitination. **(A)** mRNA expression of *ERα* of BMMs from WT and PGRN-KO mice. *n* = 4 biological replicates; significant difference was analyzed by Student's *t*-test, with significance level set to 0.05 (*P* < 0.05). Data are presented as box and whisker plots showing the median, minimum to maximum. **(B)** ERα protein expression in BMMs from WT and PGRN-KO mice determined by immunoblotting. **(C)** Immunohistochemistry staining of ERα in tibia tissue sections of WT and PGRN-KO mice. Scale bar: 500 μm. **(D)** Bone marrow cells were obtained from WT and PGRN-KO mice, and cultured into BMMs. BMMs were collected and subjected to lysis in RIPA buffer containing protease inhibitors. The lysate was immunoprecipitated with ERα antibody, and then ubiquitin, K63- and K48-linkage specific polyubiquitin were detected by immunoblotting. In B–D, *n* = 3 biological replicates. PGRN, progranulin; ERα, estrogen receptor α; BMMs, marrow-derived macrophages.

Interestingly, ERα expression in astrocytes has been reported to be significantly suppressed in PGRN-deficient mice.^29^ In the present study, we found that PGRN deficiency did not change ERα mRNA level (Fig. 7A), but reduced its protein level in BMMs (Fig. 7B). Immunohistochemistry analysis indicated that ERα expression in the bone tissue was also markedly decreased in 12-week-old PGRN-KO OVX mice than that in 12-week-old WT OVX mice (Fig. 7C). Furthermore, increased ubiquitination of ERα, especially by K48-linked ubiquitination, was observed in BMMs from PGRN-KO mice than in BMMs from WT mice (Fig. 7D), indicating that PGRN may enhance ERα degradation via K48-linked ubiquitination. In contrast, rhPGRN could increase ERα gene (*F* = 23.49; *P* < 0.001) and protein expression in Raw264.7 cells (Fig. S7).

## Discussion

A recent study indicated PGRN to be associated with osteoarthropathy.^30^ In the present study, mass spectrometry results revealed a positive correlation between hip bone PGRN expression and hip BMD in postmenopausal women. High PGRN levels were associated with high BMD, and low PGRN levels were associated with low BMD. These findings are concordant with those of a study by Bateman et al that found a significant correlation between serum PGRN levels and hip BMD in obese individuals.^7^ The results of serum parathyroid hormone and vitamin D levels were also consistent with those in a previous study.^18^ The association between PGRN and BMD can be explained by the regulatory roles of PGRN in signaling pathways. According to previous studies, PGRN may activate its receptors and antagonize TNF-α that acts as an inhibitor of osteoblast differentiation and activator of osteoclastogenesis.^31^^,^^32^

The general phenotype of PGRN-KO mice has been previously reported.^11^^,^^33^, ^34^, ^35^, ^36^ Young adult PGRN-KO mice were reported to be healthy and fertile, and the overall whole-body pathological evaluation was normal.^33^^,^^34^ The results of the present study suggest that there were no significant differences in bone mass between 12-week-old female WT and PGRN-KO mice. However, in line with previous reports, we also found that PGRN deficiency exacerbated trabecular bone loss in the OVX- and age-induced osteoporosis model.^35^^,^^36^ However, PGRN deficiency did not show significant effects on cortical bone parameters in our current OVX model, which is in line with a previous report that PGRN deficiency did not affect cortical bone in an age-induced osteoporosis model.^35^ The underlying mechanism of this discrepancy of PGRN's effect on trabecular bone and cortical bone in the osteoporosis model warrants further investigation. Furthermore, PGRN has been reported to enhance endochondral ossification during development and also act as a critical mediator of the bone healing process by modulating BMP-2 and TNF-α signaling.^14^^,^^37^^,^^38^ Therefore, the absence of significant differences in the bone mass between young WT and PGRN-KO mice may be attributed to the strong effects of estrogen on functions of PGRN and/or the functional redundancy between estrogen and PGRN in regulating bone remodeling. Taken together, in the presence of estrogen, depletion of PGRN led to a mild change in the bone mass; while in the absence of estrogen, depletion of PGRN significantly promoted osteoclastogenesis and subsequent trabecular bone loss.

Consistent with the results of a previous study,^25^ we found that estradiol stimulated PGRN gene and protein expression in a time-dependent manner in both the BMMs and Raw264.7 cells. Therefore, we hypothesized that PGRN mediates the effects of estrogen on bone metabolism. To verify this assumption, we treated OVX mice with 17β-estradiol pellets in both WT and PGRN-KO groups. It is known that implantation of pellets has an advantage over traditional intraperitoneal injection in reducing experimental error.^39^, ^40^, ^41^ The dosage selected in the present study was based on that administered in previous studies.^40^, ^41^, ^42^ Dogs and rats treated with estrogen in previous studies showed a greater increase in vertebral bone strength than control.^43^^,^^44^ In the present study, we found that estrogen increased the trabecular BMD, BV/TV, trabecular bone number, cortical thickness, cortical area, and cortical BMD in WT OVX mice, while these effects were reduced in PGRN-KO mice, suggesting that the effects of estrogen on bone metabolism were at least partially mediated by PGRN. One explanation for the continued increase in BMD, BV/TV, and trabecular bone number in the PGRN-KO mice treated with estrogen could be the persistent high dosage of 17β-estradiol pellets, as opposed to the dense microstructure in the WT OVX mice treated with estrogen.

The cell differentiation experiments indicated that the BMMs from PGRN-KO mice had an increased osteoclast formation and bone resorption, and BMSCs from PGRN-KO mice had a decreased osteogenic differentiation than BMSCs from WT mice. We found that estrogen increased osteogenic differentiation in WT BMSCs, consistent with results of previous studies,^23^^,^^45^, ^46^, ^47^, ^48^ which is independent of PGRN given that estrogen could also enhance osteogenesis in PGRN KO BMSCs. In contrast, unlike estrogen regulation of osteogenesis, its regulation of osteoclastogenesis depended on PGRN.^22^^,^^26^^,^^49^, ^50^, ^51^, ^52^, ^53^, ^54^, ^55^, ^56^, ^57^ Therefore, we concluded that PGRN is required for the effects of estrogen on bone metabolism, primarily via osteoclastogenesis, but not osteoblastogenesis. Of note, estrogen's effect on bone metabolism under the condition of PGRN overexpression warrants further investigation.

The direct effects of PGRN on osteoclasts were, however, not investigated in the present study. The physiological levels of PGRN have been reported to predominantly induce osteoblastogenesis^35^^,^^58^^,^^59^ and inhibit osteoclastogenesis.^35^^,^^60^^,^^61^ However, a study by Oh et al demonstrated that PGRN induced osteoclastogenesis in the presence of receptor activator of nuclear factor kappa-B ligand (RANKL).^62^ Therefore, the role of PGRN in osteoclasts remains controversial. Our findings support the hypothesis that PGRN acts as an antagonist of osteoclastogenesis. The inconsistency in PGRN regulation of osteoclastogenesis may result from the different amounts of PGRN used in these studies. It is well established that the normal and pathological actions of PGRN depend on PGRN levels.^9^^,^^63^

A previous study indicated that ERα expression in the astrocytes was undetected in PGRN-KO mice^29^; consistent with this finding, we found that ERα expression in the BMMs and bones was significantly lower in PGRN-KO mice than in WT mice. The ERs were reported to be present in the osteoblasts, osteocytes, and osteoclasts.^64^, ^65^, ^66^ Furthermore, ERα has also been observed in mononuclear preosteoclasts.^67^^,^^68^ As PGRN deficiency only affects ERα protein level, but not mRNA level, PGRN has been reported to be involved in the ubiquitination of C/EBPα protein 69, and ERα has been reported to be degraded through the ubiquitin-proteasome pathway.^70^^,^^71^ We hypothesized that PGRN regulated ERα expression at the post-transcriptional level. The results indeed indicated that PGRN insufficiency increased ubiquitination of ERα, primarily via K48-linked ubiquitination, but not via K63-linked ubiquitination. Of note, the molecular link between PRGN and ubiquitination of ERα warrants further investigation. In addition, the findings that estrogen induces PGRN expression (Fig. 3), PGRN also induces ERα expression (Fig. S7), and PGRN deficiency causes the reduction of ERα (Fig. 7), suggest the existence of a positive feedback regulatory loop between estrogen and PGRN signaling pathways.

In summary, the present study identified PGRN as an important factor in the pathogenesis of postmenopausal osteoporosis and confirmed that PGRN is critical to mediating the effects of estrogen on bone metabolism. Furthermore, a mechanism study indicates that loss of PGRN after menopause may promote bone loss by lowering ERα expression by increasing its ubiquitination. The study findings may also provide a new therapeutic approach for the prevention and treatment of postmenopausal osteoporosis and other estrogen/ERα associated diseases and conditions.

## Author contributions

GL, YX, and CJL designed the experiments and wrote the manuscript. GL, AW, WT, and WF performed most experiments. QD and YX assisted with the collection of femoral head samples. MW assisted with mass spectrometry data interpretation. QT, YD, and JW assisted with animal experiments. WF and JJ assisted with immunofluorescence and protein immunoprecipitation. XZ assisted with the purification of recombinant human PGRN. ML, AH, WF, and CJL assisted with analyzing the data and editing the manuscript.

## Funding

This work was supported by the grants from 10.13039/501100004543China Scholarship Council (No. 201505320002), the 10.13039/100000002National Institutes of Health (No. R01AR062207, R01AR061484, R01AR076900, R01NS103931), Young Medical Talents Program of Jiangsu Province, China (No. QNRC2016878), 10.13039/501100012166National Key R&D Program of China (No. SQ2021YFC2501702), 10.13039/501100001809Natural Science Foundation of China (No. 82072474), and Clinical Medicine Technology Project of Jiangsu Province, China (No. BE2019661).

## Conflict of interests

Chuanju Liu is the member of *Genes & Diseases* Editorial Board. To minimize bias, he was excluded from all editorial decision-making related to the acceptance of this article for publication. The remaining authors declare no conflict of interest.
