# Supplementary material for: Progranulin deficiency associates with postmenopausal osteoporosis via increasing ubiquitination of estrogen receptor α
Source: Genes Dis. 2024 Jan 28;12(1):101221. doi: 10.1016/j.gendis.2024.101221 (PMC11570241; doi:10.1016/j.gendis.2024.101221)
Supplement: Multimedia component 1 [file mmc1.pdf]

Progranulin deficiency associates with postmenopausal osteoporosis via increasing ubiquitination of estrogen receptor α

Guangfei Li, Aifei Wang, Wei Tang, Wenyu Fu, Qingyun Tian, Jinlong Jian, Michal Lata, Aubryanna Hettinghouse, Yuanjing Ding, Jianlu Wei, Xiangli Zhao, Mingyong Wang, Qirong Dong, Chuan-ju Liu, Youjia Xu

This Supplementary Materials File Includes:

Supplementary Figures S1 to S7 (Pages 2-8)

Supplementary Table1 to Table 2 (Page 9-11)

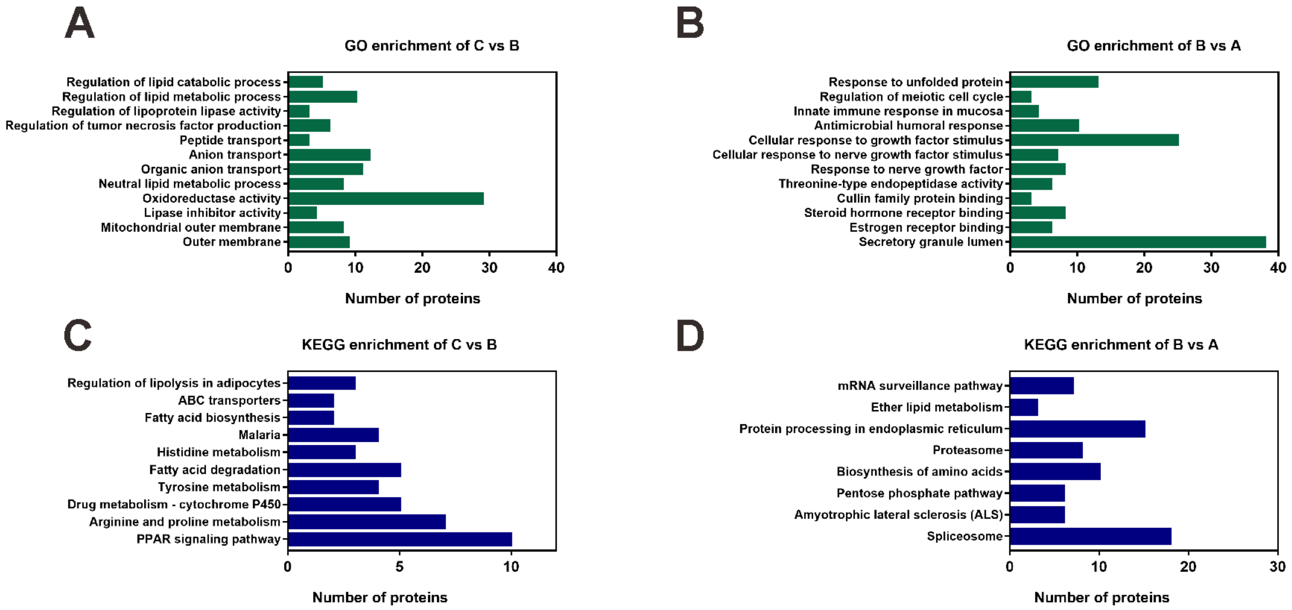

Supplementary Figure 1. Mass spectrometry of 83 femoral head specimens of postmenopausal women with one-side hip fragility fracture. (**A**-**B**) GO enrichment analysis of group C vs group B (**A**) and group B vs group A (**B**). (**C**-**D**) KEEG enrichment analysis of group C vs group B (**C**) and group B vs group A (**D**).

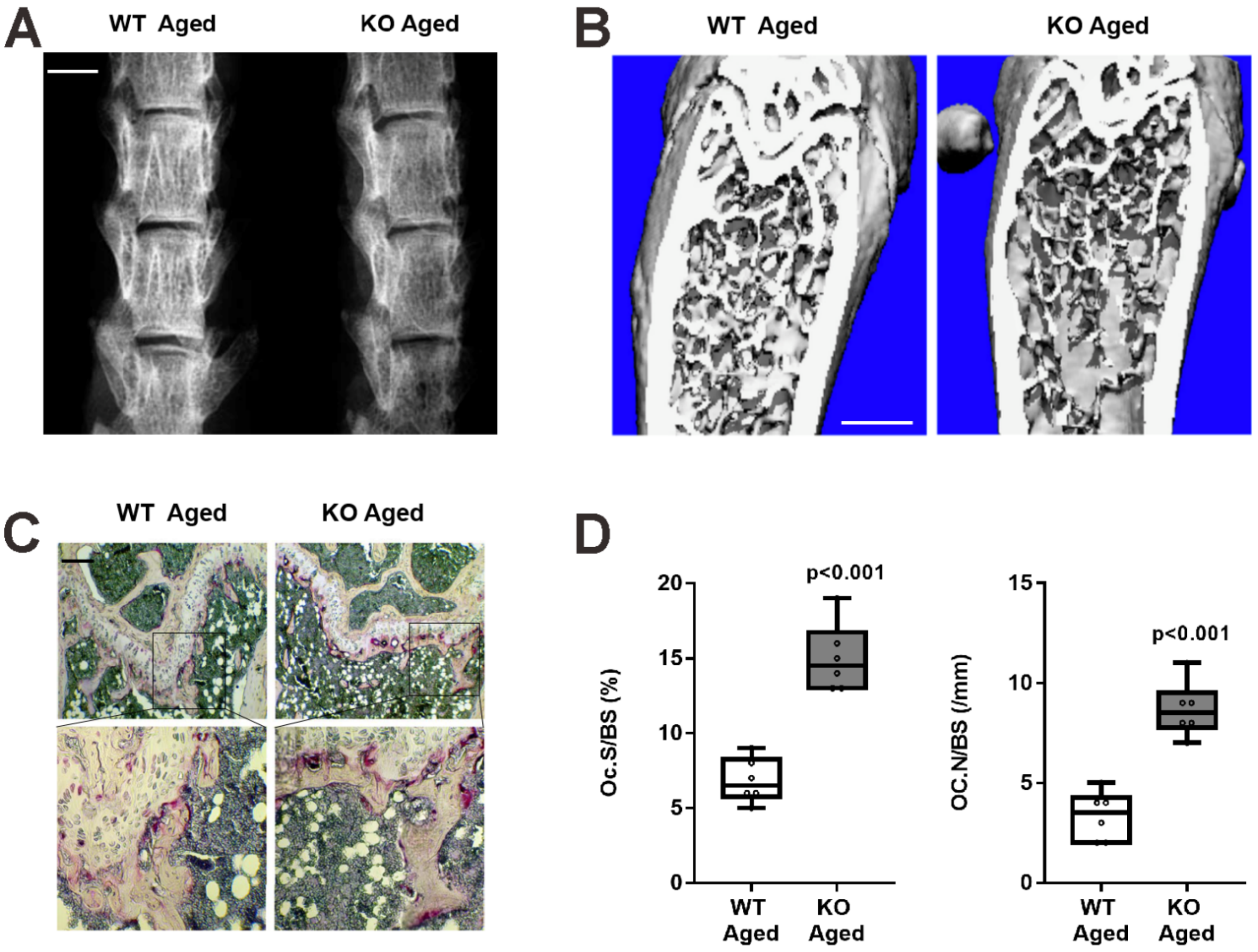

Supplementary Figure 2. PGRN deficiency exacerbates bone loss in aged osteoporosis model. (A) Micro-radiographic analysis of spines from 40-week-old WT and PGRN-KO female mice. Scale bar represents 2 mm. (**B**) Coronal micro-CT images of the distal femur from 40-week-old WT and PGRN-KO female mice. Scale bar: 1 mm. (**C-D**) TRAP staining (**C**) and quantification of Oc.N/BS and Oc.S/BS (**D**) in the proximal tibia of 40-week old WT and PGRN-KO female mice. Scale bar: 250 µm. Student's t tests compared groups, with significance noted below $p < 0.05$. $n = 6$ mice per group. Data are presented as box and whisker plots showing the median, minimum to maximum.

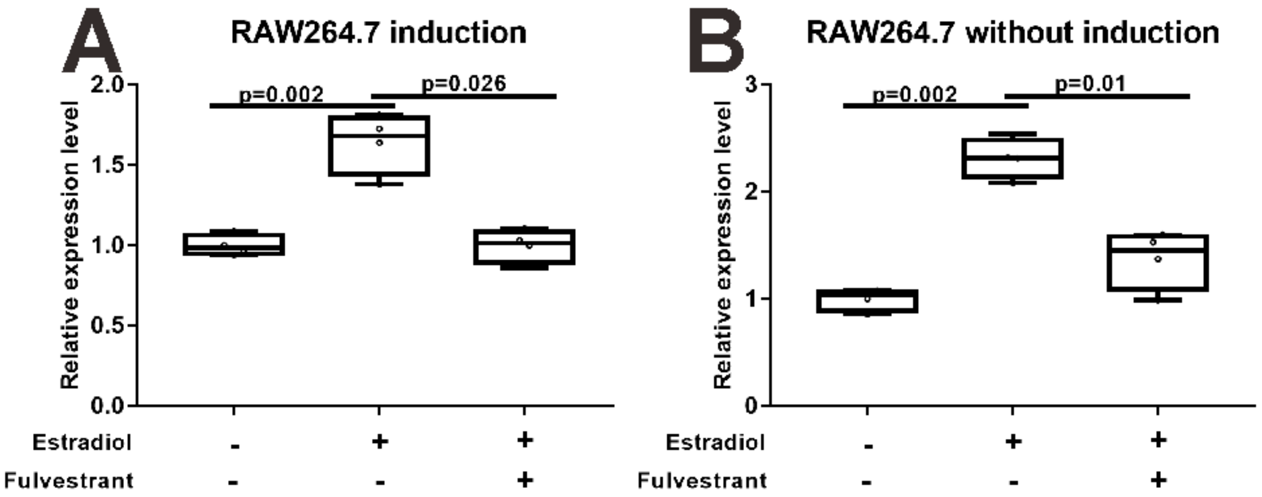

Supplementary Figure 3. Estradiol stimulates PGRN gene expression via ER in RAW 264.7 cells. (**A**) RAW 264.7 cells were treated with or without estradiol (10 nM) in the absence or presence of fulvestrant (10 nM) for 2 days in α-MEM supplemented with 50 ng/ml RANKL. (**B**) RAW 264.7 cells were treated with or without estradiol (10 nM) in absence or presence of fulvestrant (10 nM) for 2 days in α-MEM. Cells were collected, and *Grn* mRNA levels were measured by real-time PCR. In **A**-**B**, n = 4 biological replicates; significant difference was analyzed by one-way ANOVA with Bonferroni's post hoc test, with significance noted below $p < 0.05$. Data are presented as box and whisker plots showing the median, minimum to maximum.

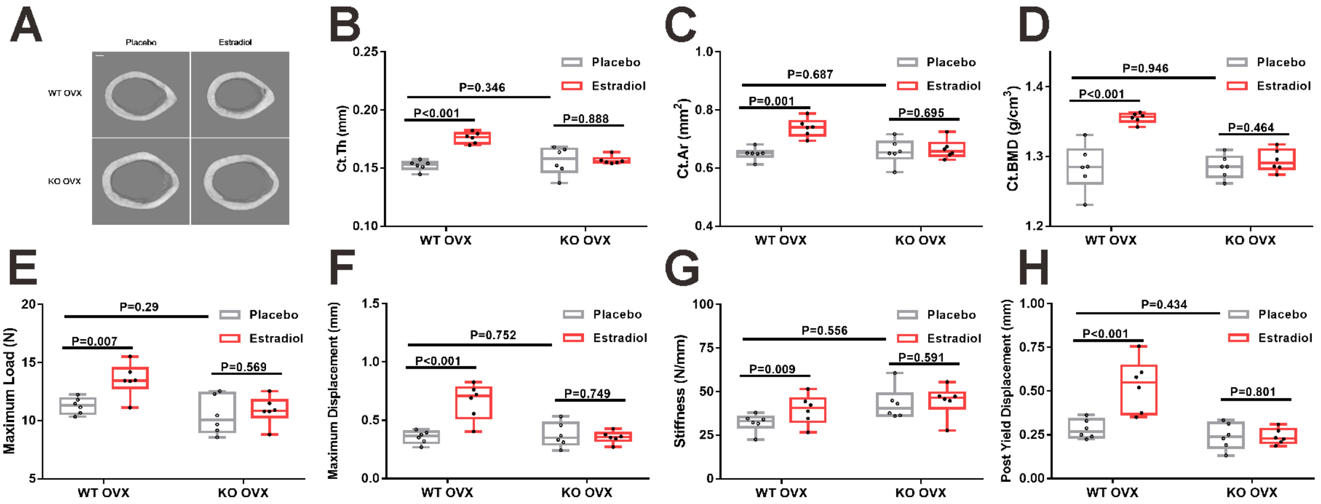

Supplementary Figure 4. Estradiol's protection on cortical bone after OVX is blunted in PGRN-KO mice. (**A**) Representative three-dimensional reconstruction of cortical bone at femur of WT OVX mice and PGRN-KO OVX mice treated with estradiol or placebo. Scale bar: 200 µm. (**B-D**) Micro-CT assessment of Ct.Th (**B**), Ct.Ar (**C**), and Ct.BMD (**D**) at femur of WT OVX mice and PGRN-KO OVX mice treated with estradiol or placebo. (**E-H**) Biomechanical testing of femur Maximum Load (**E**), Maximum Displacement (**F**), Stiffness (**G**), and Post Yield Displacement (**H**) of WT OVX mice and PGRN-KO OVX mice treated with estradiol or placebo. In **A-H**, n = 6 mice per group. In **B-H**, significant difference was analyzed by one-way ANOVA with Bonferroni's post hoc test. Data are presented as box and whisker plots showing the median, minimum to maximum.

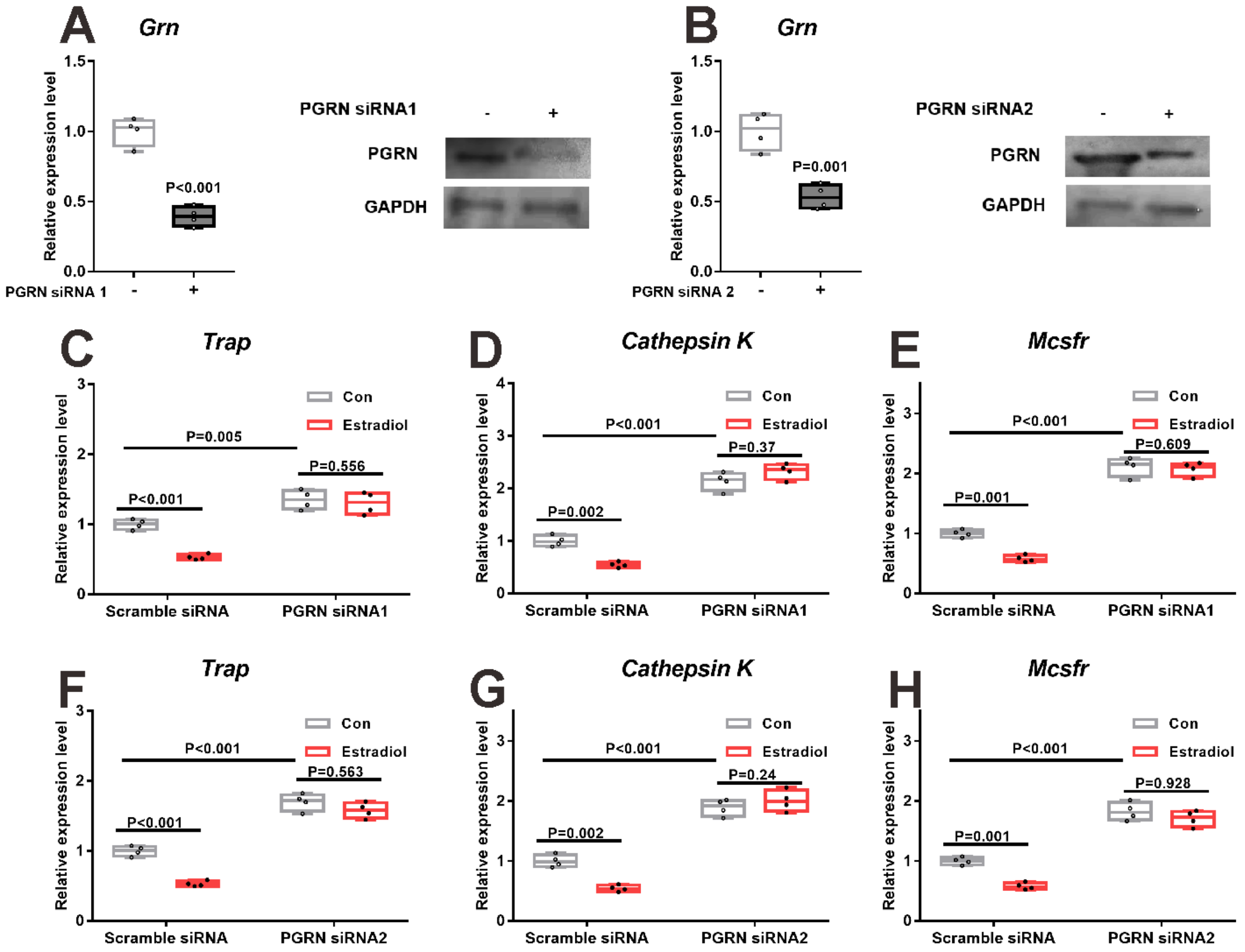

Supplementary Figure 5. Effects of estradiol on osteoclastic gene expression in PGRN knockdown Raw264.7 cells. (**A-B**) Knockdown efficiency of PGRN by PGRN siRNA1 (**A**) and siRNA2 (**B**) in Raw264.7 cells measured by qRT-PCR and Western blot. (**C-E**) Fold change of osteoclast differentiation markers *Trap* (**C**), *Cathepsin K* (**D**), and *Mcsfr* (**E**) mRNA in Raw264.7 cells transfected with scramble or PGRN siRNA1 under differentiation medium in the presence or absence of estradiol for 48h. (**F-H**) Fold change of osteoclast differentiation markers *Trap* (**F**), *Cathepsin K* (**G**), and *Mcsfr* (**H**) mRNA in Raw264.7 cells transfected with scramble or PGRN siRNA2 under differentiation medium in the presence or absence of estradiol for 48h. In **A-B**, n = 4 biological replicates; significant difference was analyzed by un-paired Student's t test, with significance noted below $p < 0.05$. In **C-H**, n = 4 biological replicates; significant difference was analyzed by one-way ANOVA with Bonferroni's post hoc test. "Con" indicates control group treated with PBS. Data are presented as box and whisker plots showing the median, minimum to maximum.

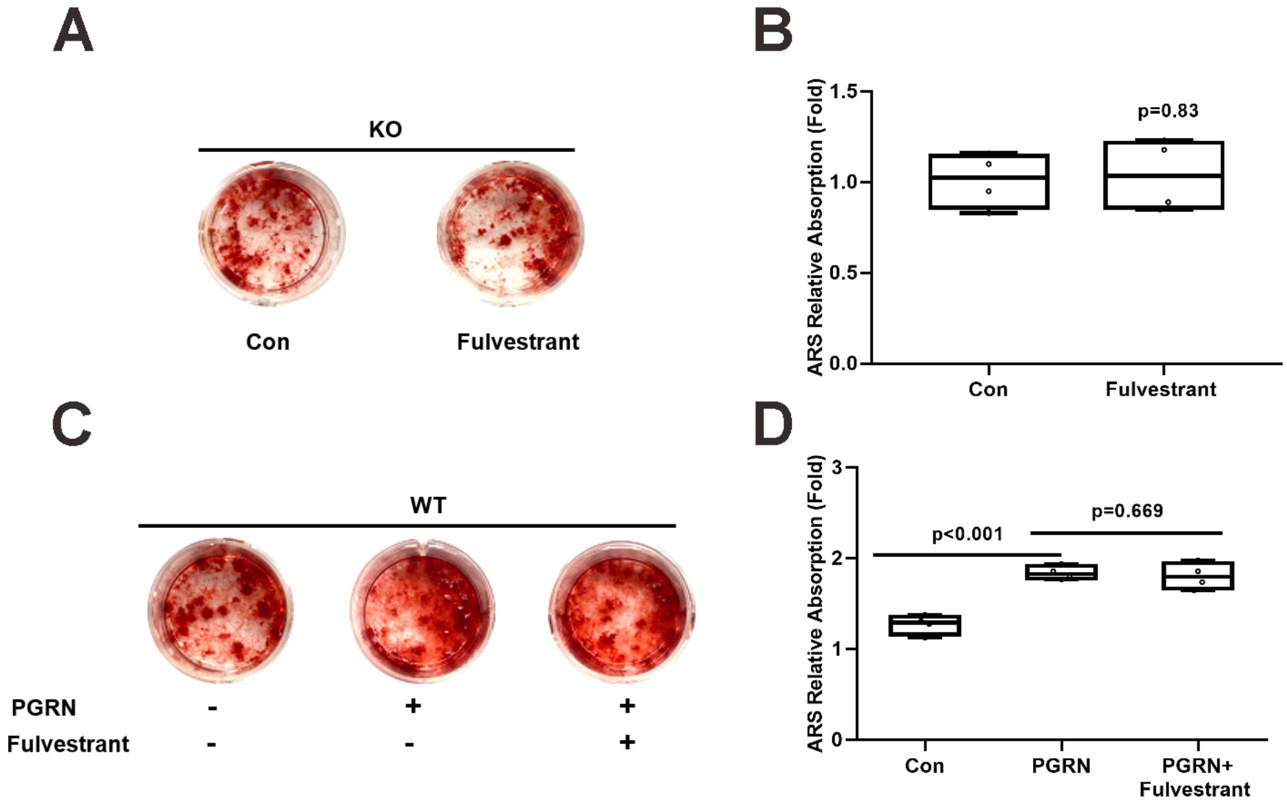

Supplementary Figure 6. rhPGRN ehnances osteoblast differentiation independent of ER. (**A-B**) BMSCs from PGRN-KO mice were treated with or without fulvestrant (10 nM) under osteogenic differentiation medium. ARS staining (**A**) were performed at 3 weeks, the intensity (**B**) of ARS staining was quantified with 10% CPC. (**C-D**) BMSCs from WT mice were treated with or without PGRN (200 ng/ml) in the absence or presence of fulvestrant (10 nM) under osteogenic differentiation medium. ARS staining (**C**) were performed at 3 weeks, the intensity (**D**) of ARS staining was quantified with 10% CPC. In **A** and **C**, n = 4 biological replicates, representative images were shown. In **B** and **D,** n = 4 biological replicates; significant difference was analyzed by unpaired Student's t test (B) and one-way ANOVA with Bonferroni's post hoc test (D), with significance noted below $p < 0.05$. "Con" indicates control group treated with PBS. Data are presented as box and whisker plots showing the median, minimum to maximum.

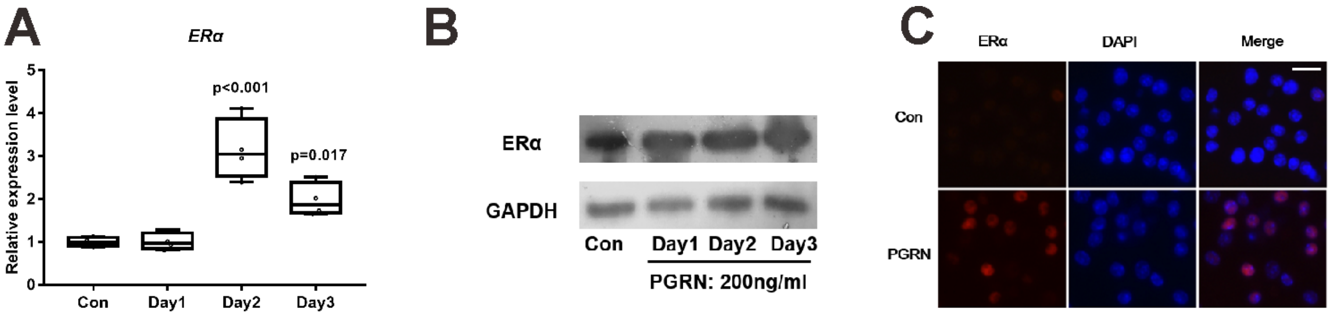

Supplementary Figure 7. rhPGRN treatment increases ERα gene and protein expression in Raw264.7 cells. (**A**) Raw264.7 cells were treated with recombinant human PGRN (200 ng/ml) for 3 days. Cells were collected, and *ERα* mRNA levels were measured by real-time PCR. rhPGRN treated groups (n = 4 for each time point) were compared with the control group (n = 4) using a Dunnett's test, with significance noted below $p < 0.05$. Data are presented as box and whisker plots showing the median, minimum to maximum. (**B**) Raw264.7 cells were treated with recombinant human PGRN (200 ng/ml) for 3 days, and ERα protein levels were determined by Immunoblotting. n = 3 biological replicates and representative images were shown. (**C**) Raw264.7 cells were treated with recombinant human PGRN (200 ng/ml) for 3 days, then cells were fixed and stained with ERα primary antibody and fluorescent secondary antibody. Scale bar: 25 µm. n = 3 biological replicates and representative images were shown. "Con" indicates control group treated with PBS.

Supplementary Table 1: Primers used for quantitative RT-PCR

**Gene**	**Primers** (Forward/Reverse)
Grn	(F) 5'- CTGCCCGTTCTCTAAGGGTG -3'
	(R) 5'- ATCCCCACGAACCATCAACC -3'
Mcsfr	(F) 5'- GGTGGCTGTGAAGATGCTAA -3'
	(R) 5'- AGGTCCTCCGTGAGTACAGG -3'
Nfatc1	(F) 5'- ATACCTGGCTCGGTAACACC -3'
	(R) 5'- CATGCTCCAGTGCTGTCTTT -3'
Cathepsin K	(F) 5'- CAGCAGAACGGAGGCATTGA -3'
	(R) 5'- CTTTGCCGTGGCGTTATACATACA -3'
Trap	(F) 5'- TTGCGACCATTGTTAGCCACATA -3'
	(R) 5'- TCAGATCCATAGTGAAACCGCAAG -3'
Runx2	(F) 5'- TCGGAGAGGTACCAGATGGG -3'
	(R) 5'- AGGTGAAACTCTTGCCTCGT -3'
Alp	(F) 5'- GCTGATCATTCCCACGTTTT -3'
	(R) 5'- ACCATATAGGATGGCCGTGA -3'
Osx	(F) 5'- GCTCGTAGATTTCTATCCTC -3'
	(R) 5'- CTTAGTGACTGCCTAACAGAGA -3'
Col1	(F) 5'- TGACTGGAAGAGCGGAGAGTA -3'
	(R) 5'- GACGGCTGAGTAGGGAACAC -3'
ERα	(F) 5'- AATTCTGACAATCGACGCCAG-3'
	(R) 5'- GTGCTTCAACATTCTCCCTCCTC-3'
Gapdh	(F) 5'- AGAACATCATCCCTGCATCC -3'
	(R) 5'- AGTTGCTGTTGAAGTCGC -3'

Supplementary Table 2

Detailed baseline characteristics of all the sample's corresponding clinical information by group

Variables	Normal (A)	Osteopenia (B)	Osteoporosis (C)	*F*-Value	*p*-Value
Femoral neck T-score	T ≥ -1.0	-2.5 < T < -1.0	T ≤ -2.5		
**Samples information**					
Total number of samples	5	46	32		
Ordinal sample numbers	S1, S19, S46, S49, S60	S2, S5-8, S10-14, S16-17, S20-25, S27-29, S33, S37-38, S40-41, S43, S45, S50, S53, S55-56, S58, S62-63, S67, S69-70, S76-77, S80-81, S84, S92-94	S15, S18, S26, S30-31, S34, S36, S39, S42, S47, S51-52, S54, S57, S59, S64, S66, S71-75, S78, S82, S85-91, S95		
**Anthropometry**					
Age (years)	79.20 ± 13.83	73.04 ± 7.97 (p=0.144)	80.87 ± 6.81 (p=0.798)	9.468	p<0.001
Height (cm)	156.00 ± 4.18	156.63 ± 4.97 (p=0.901)	153.19 ± 4.30 (p=0.286)	5.168	p=0.008
Weight (Kg)	49.00 ± 7.38	56.66 ± 8.16 (p=0.061)	48.33 ± 7.49 (p=0.959)	11.259	p<0.001
BMI (Kg/cm^2)	20.21 ± 3.49	23.07 ± 3.01 (p=0.068)	20.58 ± 3.24 (p=0.919)	7.295	p=0.001
**Bone densitometry**					
Femoral neck T-score	0.46 ± 1.03	-1.81 ± 0.41 (p<0.001)	-3.23 ± 0.65 (p<0.001)	124.796	p<0.001
Femoral neck BMD (g/cm^2)	0.98 ± 0.12	0.72 ± 0.55 (p<0.001)	0.55 ± 0.83 (p<0.001)	102.392	p<0.001
Lumbar T-score	-1.58 ± 0.92	-1.76 ± 1.10 (p=0.842)	-2.68 ± 0.94 (p=0.043)	8.153	p=0.001
Lumbar BMD (g/cm^2)	0.92 ± 0.11	0.90 ± 0.14 (p=0.863)	0.79 ± 0.11 (p=0.037)	8.984	p<0.001
Greater trochanter BMD (g/cm^2)	0.82 ± 0.37	0.61 ± 0.06 (p<0.001)	0.47 ± 0.08 (p<0.001)	29.362	p<0.001

Total hip BMD (g/cm^2)	0.93 ± 0.09	0.76 ± 0.09 (*p*<0.001)	0.58 ± 0.09 (*p*<0.001)	53.978	*p*<0.001
**Biochemistry characteristics**					
PTH (pg/ml)	55.40 ± 20.51	72.69 ± 76.57 (*p*=0.816)	66.90 ± 37.25 (*p*=0.907)	0.105	*p*=0.900
Vitamin D (nmol/L)	32.04 ± 10.91	36.63 ± 10.84 (*p*=0.615)	29.51 ± 7.10 (*p*=0.835)	3.163	*p*=0.052
Hb (g/L)	104.40 ± 18.20	123.11 ± 14.49 (*p*=0.015)	115.44 ± 15.23 (*p*=0.177)	5.006	*p*=0.009
Serum calcium (mmol/L)	2.18 ± 0.11	2.22 ± 0.13 (*p*=0.662)	2.17 ± 0.12 (*p*=0.927)	1.618	*p*=0.205
Serum phosphorus (mmol/L)	1.03 ± 0.21	1.09 ± 0.21 (*p*=0.713)	1.08 ± 0.21 (*p*=0.752)	0.153	*p*=0.858
Serum uric acid (µmol/L)	339.80 ± 55.76	272.63 ± 108.12 (*p*=0.239)	292.22 ± 105.28 (*p*=0.448)	1.073	*p*=0.347
ALB (g/L)	39.08 ± 5.82	40.23 ± 3.21 (*p*=0.649)	37.43 ± 4.30 (*p*=0.475)	5.073	*p*=0.008
ALP (U/L)	86.60 ± 22.51	80.67 ± 22.57 (*p*=0.736)	82.25 ± 26.37 (*p*=0.842)	0.153	*p*=0.858
CRP (mg/L)	25.78 ± 33.41	30.17 ± 30.42 (*p*=0.888)	39.62 ± 29.94 (*p*=0.445)	1.086	*p*=0.342
PINP (ng/mL)	64.46 ± 48.13	58.18 ± 44.37 (*p*=0.872)	59.38 ± 29.54 (*p*=0.915)	0.058	*p*=0.944
β-CTX (pg/ml)	536.66 ± 130.85	503.80 ± 357.18 (*p*=0.953)	698.71 ± 378.13 (*p*=0.453)	2.647	*p*=0.078

BMI, body mass index; BMD, bone mineral density; PTH, parathyroid hormone; Hb, hemoglobin; ALB, albumin; ALP, alkaline phosphatase; CRP, C-reactive protein; PINP, type I procollagen amino-terminal peptide; β-CTX, β-type I collagen carboxy-terminal peptide.

Significant difference was analyzed by one-way ANOVA with F-value and *p*-value. Osteopenia (B) and Osteoporosis (C) groups were compared with the Normal (A) group using a Dunnett's test, (*p)* indicated the actual *p* values.
