# Supplementary material for: Progranulin deficiency associates with postmenopausal osteoporosis via increasing ubiquitination of estrogen receptor α
Source: Genes Dis. 2024 Jan 28;12(1):101221. doi: 10.1016/j.gendis.2024.101221 (PMC11570241; doi:10.1016/j.gendis.2024.101221)
Supplement: Multimedia component 2 [file mmc2.pdf]

Supplemental file-1 - All the sample's corresponding clinical information

Sample Number	Age (years)	Height （cm）	Weight （Kg）	BMI	Femoral Neck T-score	Femoral Neck BMD （g/cm2）	Lumbar T-score	Lumbar BMD （g/cm2）	Greater trochanter BMD （g/cm2）	Total hip BMD （g/cm2）	PTH （pg/mL）	PINP (ng/mL)	β-CTX (pg/ml)	Vitamin D (nmol/L)	Hb (g/L)	Serum calcium (mmol/L)	Serum phosphorus (mmol/L)	Serum uric acid (μmol/L)	ALB (g/L)	ALP (U/L)	CRP (mg/L)
S1	58	160	40	15.63	0.6	1.004	-2.1	0.863	0.702	0.977	-	36	482.8	-	135	2.19	0.74	300	46	104	7
S19	79	150	55	24.44	1.7	1.131	-1.7	0.91	0.673	0.903	-	147.8	752.8	-	100	2.37	1.29	299	44.7	90	6.3
S46	95	155	43	17.90	1.1	1.059	-0.3	1.076	1.469	1.036	69.9	27.82	549.6	24.33	86	2.13	0.9	348	35.9	64	13.6
S49	87	160	57	22.27	-0.9	0.82	-1.1	0.978	0.535	0.796	-	55.64	490	-	101	2.11	1.1	433	35.4	63	85
S60	77	155	50	20.81	-0.2	0.904	-2.7	0.791	0.698	0.955	40.9	55.02	408.1	39.76	100	2.11	1.12	319	33.4	112	17
S2	72	159	69	27.29	-1.6	0.738	-2.2	0.846	0.621	1.133	-	39.52	429.6	-	117	2.07	1.03	247	42.8	117	7
S5	77	150	45	20.00	-1.7	0.721	-0.1	1.102	0.703	0.848	-	26.14	534	-	115	2.07	1.06	287	41.3	57	28.3
S6	85	155	65	27.06	-2.3	0.657	-2.5	0.82	0.597	0.723	-			-	122	2.15	0.74	374	39.5	77	64.8
S7	74	150	59	26.22	-2.2	0.823	-0.5	1.057	0.668	0.975	-	36.15	243.6	-	140	2.3	1.16	378	47.2	80	10.2
S8	57	168	68.5	24.27	-1.6	0.738	-1.7	0.909	0.646	0.806	-	67.91	1280	-	125	2.25	1.16	166	42.9	101	19.7
S10	74	162	55	20.96	-2.3	0.655	-2.5	0.816	0.681	0.799	-			42.7	122	2.23	0.88	122	42.4	113	5
S11	65	156	63	25.89	-2.1	0.674	-1.6	0.926	0.576	0.736	-	29.53	132.7	21.5	131	2.31	1.06	324	42.4	99	16
S12	63	153	50	21.36	-2.2	0.671	-0.7	1.027	0.609	0.76	-	57.04	651.8	-	121	2.03	0.88	197	39.2	60	33.9
S13	62	165	54	19.83	-1.5	0.744	-1.5	0.933	0.591	0.777	45.9	41.29	510.2	37.41	149	2.35	1.11	138	44.8	85	9
S14	52	162	52.5	20.00	-1.8	0.715	-2.2	0.856	0.637	0.787	51.8	42.63	266.3	32.97	131	2.24	0.97	181	42.4	63	10.6
S16	72	163	69	25.97	-1.3	0.779	-1.6	0.922	0.7	0.837	66.1	43.63	223.2	50.96	144	2.47	1.42	385	43.3	80	29.8
S17	75	150	60	26.67	-2.2	0.661	-2.3	0.833	0.586	0.712	58.4	45.9	556	36.53	120	2.1	0.94	177	38.6	88	45
S20	72	160	68	26.56	-1.2	0.786	0.2	1.133	0.663	0.853	-	35.32	519	-	126	2.12	1.21	267	34.3	83	77.2
S21	70	156	48	19.72	-2.2	0.66	-3.6	0.686	0.512	0.678	-	27.89	148	-	129	2.18	0.97	206	43.7	61	6.7
S22	74	166	60	21.77	-1.9	0.706	-2	0.873	0.533	0.734	-	63.88	353.7	-	148	2.29	1.14	283	36.9	102	16
S23	73	156	56	23.01	-1.7	0.726	-1.5	0.935	0.659	0.811	-	71.75	306.8	-	84	2.21	1.2	423	40.6	92	7.2
S24	70	152	70	30.30	-1.7	0.73	0.1	1.125	0.552	0.711	-	104.6	407	-	117	2.16	1.15	579	37.1	62	7.6
S25	82	158	55	22.03	-1.3	0.774	-1	0.995	0.672	0.791	-	73.24	893.8	-	124	2.11	1.07	180	36.3	154	26.3
S27	79	160	54	21.09	-1.5	0.746	-2.5	0.819	0.571	0.724	-	17.08	144.1	-	94	2.33	0.87	121	44.5	69	26.9
S28	68	159	60	23.73	-1.2	0.789	0.8	1.214	0.708	0.813	-	9.34	159.3	-	137	2.19	1.24	389	43.2	72	48.1
S29	79	145	43	20.45	-2.1	0.68	-2.9	0.77	0.558	0.697	67	73.82	959.1	42.18	117	2.25	1.11	225	37.3	65	5.5
S33	71	155	50	20.81	-1.6	0.792	-2.2	0.756	0.552	0.696	85	53.07	961.7	45.51	128	2.19	1.06	271	39	64	5.7
S37	69	153	60	25.63	-1.3	0.776	-0.4	1.07	0.601	0.81	81.8	49.44	502.5	41.47	120	2.3	1.04	438	42	77	32.7
S38	79	156	55	22.60	-2.4	0.647	-1.9	0.884	0.559	0.678	242.7	43.44	957.7	27.34	127	2.46	0.93	251	42.9	103	18.6
S40	58	150	48.5	21.56	-1.8	0.718	-2.4	0.829	0.616	0.777	29.8	74.36	466.8	33.82	134	2.43	1.17	251	46	94	19.4
S41	75	158	48	19.23	-1.3	0.775	-1.6	0.927	0.646	0.907	342.5	127	1969	30.45	107	2.63	2.13	442	36.9	52	50.1
S43	77	158	56	22.43	-1.8	0.717	-2	0.878	0.735	0.86	44.6	43.41	402.3	29.38	130	2.22	0.89	269	37.6	80	5.6
S45	77	163	65	24.46	-2.3	0.648	-1.5	0.935	0.531	0.665	33.2	279.6	671.1	26.3	100	2.39	1.22	452	41	90	31.4
S50	83	160	65	25.39	-2.4	0.642	-0.4	1.061	0.534	0.651	31.9	25.08	112.7	48.4	142	2.3	0.98	250	35.5	71	6.6
S53	73	155	57	23.73	-2	0.685	-2.9	0.762	0.563	0.698	65.8	52.62	658.9	33.77	130	2.21	0.91	230	42.1	64	22.7
S55	77	155	36	14.98	-2.4	0.64	-4.2	0.604	0.475	0.614	34.4	58.98	275.7	40.52	119	2.18	1.01	271	37.8	67	7
S56	72	158	60	24.03	-1.7	0.732	-2.5	0.81	0.608	0.746	41.5	54.47	188.2	49.97	133	2.23	0.94	247	43.7	125	6.6
S58	60	153	55	23.50	-1.2	0.813	-2.1	0.82	0.663	0.786	43	69.01	693.2	24.73	145	2.22	0.98	174	43.7	74	7.5
S62	85	162	54	20.58	-1.7	0.73	-1.2	0.968	0.602	0.747	39.3	70.52	727.2	20.73	112	2.06	1.18	199	39.2	90	57.8
S63	84	152	48	20.78	-2.2	0.669	-1.7	0.903	0.548	0.706	-	-	-	-	117	2.21	1.09	277	38.8	82	40.8
S67	73	158	72	28.84	-1.1	0.832	-1.6	0.88	0.678	0.806	28.9	139.9	454.1	40.16	106	2.12	0.91	164	34.7	62	51.4
S69	62	160	60	23.44	-1.5	0.758	-0.8	1.15	0.552	0.734	58.1	34.3	301	25.17	129	2.25	1.26	302	39.7	62	50.7

S70	66	163	64	24.09	-1.7	0.724	-1.9	0.887	0.637	0.78	34.8	38.79	720.2	30.83	138	2.35	1.13	146	37.8	49	125.3
S76	76	150	58	25.78	-2.4	0.643	-2.9	0.762	0.551	0.648	-	31.94	269.8	-	113	2.07	0.87	324	41.2	79	132.9
S77	83	155	60	24.97	-1.1	0.804	-2	0.869	0.588	0.788	-	29.89	598.7	-	114	2.15	0.88	251	41.7	123	27.3
S80	85	155	55	22.89	-1.8	0.723	-3.6	0.68	0.558	0.697	-	64.69	447.1	-	138	2.28	1.19	481	41.1	90	30.5
S81	78	150	44	19.56	-1.9	0.751	-2.2	0.88	0.608	0.716	-	96.12	304.3	-	122	2.07	1.01	156	38.7	86	7.1
S84	82	150	42	18.67	-1.8	0.768	-2.3	0.89	0.625	0.736	-	30.21	220	-	95	1.97	0.97	174	33.6	62	105.1
S92	70	158	50	20.03	-2.4	0.641	-3.9	0.642	0.502	0.602	-	-	-	66.3	121	2.12	1.32	161	36.9	95	7
S93	68	158	60	24.03	-1.5	0.745	-1.3	0.959	0.728	0.842	-	47.02	423.9	-	130	2.15	1.23	276	41.4	48	27.2
S94	82	155	60	24.97	-2.3	0.659	0.2	1.141	0.622	0.749	-	23.15	115.5	-	100	2.08	1.26	435	37.1	42	9.9
S15	75	155	55	22.89	-2.7	0.606	-1.9	0.89	0.535	0.647	60.6	69.46	654	-	136	2.3	0.9	260	43.2	95	6.9
S18	73	150	40	17.78	-3	0.567	-2.9	0.771	0.557	0.646	59.5	31.15	403.6	33.8	112	2.36	1.21	310	41.4	60	34.6
S26	57	160	51	19.92	-2.6	0.622	-2.7	0.795	0.568	0.722	-	35.06	336.6	-	130	2.16	1.14	228	41.6	49	46.8
S30	82	152	39	16.88	-2.6	0.621	-3.9	0.649	0.409	0.545	28.95	49.69	1055	28.95	123	2.25	1.12	428	41.6	62	5.5
S31	83	158	40	16.02	-4.1	0.441	-3.7	0.666	0.356	0.436	164.6	64.23	186	42.78	91	1.87	0.89	234	32.1	48	27.6
S34	84	150	45	20.00	-3.2	0.548	-1.2	0.972	0.417	0.548	62.2	83.64	1059	20.04	115	2.23	1.28	360	34.5	132	15.1
S36	85	150	34	15.11	-3.8	0.47	-3.8	0.663	0.376	0.477	66.3	30.7	503.4	26.26	113	2.23	1.09	205	43.4	97	51.8
S39	85	155	50	20.81	-2.7	0.601	-3.1	0.737	0.547	0.636	43.7	40.31	333.4	30.37	126	2.06	0.99	230	32.5	68	24.9
S42	84	160	62.5	24.41	-2.7	0.608	-2.2	0.851	0.558	0.71	67	42.89	430.9	18.24	116	2.21	1.17	219	38.8	62	59.2
S47	85	150	50	22.22	-3.9	0.459	-1	0.996	0.553	0.584	78.7	47.87	381.5	31.33	87	2.1	1.08	348	33.3	44	7.6
S51	75	145	49	23.31	-3.2	0.544	-4.3	0.598	0.48	0.584	31	28.15	716.3	31.41	136	2.29	0.93	192	44.8	69	10.3
S52	86	150	44	19.56	-2.6	0.628	-1.8	0.82	0.647	0.749	40.5	107	697.9	40.52	94	2.06	1.2	263	38.6	98	63.9
S54	78	154	45	18.97	-5.5	0.275	-1.6	0.923	0.419	0.454	43.3	53.09	684.9	24.17	125	1.99	0.77	217	33.5	76	127.3
S57	84	156	70	28.76	-3.3	0.531	-1.5	0.93	0.447	0.539	100.9	29.4	373.9	24.97	126	2.03	0.6	169	33.5	66	99.7
S59	75	150	50	22.22	-3.1	0.554	-2.8	0.775	0.458	0.558	51	45.41	883.8	43.32	130	2.19	1.24	390	39.4	66	52.9
S64	88	150	50	22.22	-3.8	0.468	-3.9	0.646	0.378	0.504	27.8	124.8	1164	25.87	104	2.25	1.22	269	29.4	125	66.7
S66	89	151	50	21.93	-2.6	0.716	-1.5	0.92	0.556	0.725	42.9	45.57	441.6	21.09	118	2.2	0.91	304	34.4	86	46.7
S71	70	161	45	17.36	-3.3	0.528	-3.2	0.733	0.438	0.538	101.7	113	1078	29.53	122	2.28	1.13	184	40.3	147	63
S72	68	160	50	19.53	-3.2	0.543	-4.4	0.587	0.409	0.552	-	130.2	1734	-	128	2.29	1.37	308	37.9	123	6.5
S73	83	152	51	22.07	-4.2	0.43	-3.4	0.705	0.419	0.482	-	28.88	840.2	-	113	2.17	0.87	271	39	60	80.2
S74	82	152	55	23.81	-3.3	0.549	-1.6	0.862	0.446	0.586	65	65.2	293.4	-	145	2.22	0.72	357	38.6	107	68.1
S75	81	153	56	23.92	-2.9	0.622	-1.7	0.835	0.58	0.637	158.5	40.1	747.2	26.63	114	2.04	1.39	452	39.9	105	46
S78	79	150	50	22.22	-2.7	0.609	-2.3	0.842	0.413	0.608	-	61.36	800.6	-	129	2.24	1.29	360	40.2	64	6.9
S82	88	150	40	17.78	-3.8	0.628	-4	0.612	0.41	0.587	-	63.98	1208	-	108	2.18	1.46	414	34.4	67	54.1
S85	81	155	45	18.73	-3.5	0.515	-3	0.755	0.422	0.523	-	79.62	1441	-	116	2.33	0.86	156	37.3	107	7
S86	87	155	59	24.56	-3.6	0.495	-2.9	0.763	0.402	0.493	51	100.2	870.1	34.83	130	2.22	1.28	311	36.8	91	7.1
S87	81	160	55	21.48	-2.5	0.636	-3	0.753	0.605	0.723	-	40.38	290	-	121	2.09	1.16	217	36.7	83	26.2
S88	83	158	45	18.03	-2.6	0.617	-2.3	0.835	0.601	0.743	59.8	45.49	595.3	23.42	112	2.13	1.03	264	37.3	69	10.6
S89	84	150	46	20.44	-3.3	0.562	-2.6	0.803	0.386	0.516	-	-	-	32.7	95	2.29	1.28	250	36.1	106	53
S90	89	145	40	19.02	-3.5	0.511	-2.9	0.77	0.409	0.585	-	52.3	429.9	-	95	2.07	1.04	341	38.3	54	18.4
S91	82	150	45	20.00	-2.5	0.625	-2.5	0.82	0.392	0.481	-	-	-	-	89	1.94	1.23	671	25.7	82	32.3
S95	82	155	40	16.65	-3.3	0.536	-2.2	0.851	0.429	0.529	-	32.18	327.9	-	95	2.09	0.75	169	43.2	64	40.9

-indicates the patient didnot have this test.

BMI: body mass index; BMD: bone mineral density; PTH: parathyroid hormone; Hb: hemoglobin; ALB: albumin; ALP: alkaline phosphatase; CRP: C-reactive protein;

PINP: type I procollagen amino-terminal peptide;β-CTX: β-type I collagen carboxy-terminal peptide
